# Diastereomeric Monomers Enable Anion‐Exchange Membranes With Controlled Local Polymer Backbone Flexibility and High Conductivity

**DOI:** 10.1002/advs.76588

**Published:** 2026-07-14

**Authors:** Si Chen, Xuchen Lyu, Triet Nguyen Dai Luong, Patric Jannasch

**Affiliations:** ^1^ Department of Chemistry Lund University Lund Sweden

**Keywords:** anion‐exchange membrane electrolyzers and fuel cells, diastereomeric compounds, hydrogen production, local chain flexibility, structure‐property relationships

## Abstract

Multiple interdependent properties govern the performance of ion‐exchange membranes. Here, we demonstrate a polymer backbone design that allows a constrained local flexibility, suppressing excessive hydration while facilitating high hydroxide conductivity in poly(arylene piperidinium) anion‐exchange membranes (AEMs). This structural design builds on a pair of diastereomeric arene monomers with vicinal methyl substitutions that introduce local conformational constraints and enable systematic investigation of how subtle stereochemical variations influence hydration behavior, ion transport, and device performance without altering the overall polymer composition. Two corresponding membrane series are synthesized that differ only in stereochemistry, providing a composition‐conserved platform for studies of structure–property relationships. The diastereomeric units in the AEMs facilitate the formation of ion‐conducting domains, providing membranes combining high hydroxide conductivity with controlled water uptake. Hence, the *syn*‐enriched membranes consistently exhibit higher water uptake than their *anti*‐enriched counterparts at comparable ion‐exchange capacities, resulting in higher hydroxide conductivity and improved anion exchange membrane water electrolysis performance. In comparison, the *anti*‐enriched membranes display improved mechanical robustness and higher hydration efficiency for ion transport. Overall, this work establishes a stereochemistry‐based synthetic strategy that enables systematic investigation and control of membrane properties through minimal structural variation, providing a molecular platform for rational AEM design.

## Graphical abstract

Stereochemistry enables control of ion‐exchange membrane properties via local backbone flexibility without altering the polymer composition. *Syn*‐enriched membranes show higher water uptake and improved water‐electrolysis performance, whereas *anti*‐enriched membranes exhibit higher hydration efficiency and improved mechanical properties.

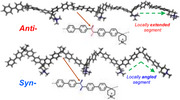

## 1 Introduction

Anion exchange membranes (AEMs) are key components in a wide range of electrochemical energy conversion and storage technologies, including anion exchange membrane fuel cells (AEMFCs), anion exchange membrane water electrolyzers (AEMWEs), redox‐flow batteries, and electrochemical CO_2_ conversion and capture systems [1, 2]. Substantial resources are currently invested to develop these technologies to address the pressing energy and climate challenges [3, 4]. Over the past decade, significant progress has been achieved through the development of chemically robust cationic moieties and ether‐free polymer backbones, which has quite dramatically improved both the alkaline stability and the ionic conductivity of AEMs [1, 2, 5, 6, 7, 8, 9, 10, 11, 12, 13, 14, 15, 16]. As a result, current research efforts are increasingly shifting from demonstrating basic material feasibility toward the rational tuning of membrane properties to satisfy the specific, and often competing requirements, imposed by different electrochemical technologies [1, 2, 15].

Besides ionic conductivity and alkaline stability, the practical usefulness of AEMs is governed by a broader set of interdependent properties, including ion‐exchange capacity (IEC), swelling behavior and water/solution uptake, thermal and mechanical stability, and gas permeability. These parameters are intrinsically coupled, such that improving one property will often unavoidably change other ones. A representative and widely encountered example is that increasing IEC values generally lead to higher water uptake and enhanced ionic conductivity [15, 16]. Although high ionic conductivity is desirable for most electrochemical applications, excessive water uptake is highly detrimental in systems with stringent water‐management requirements. In anion exchange membrane fuel cells (AEMFCs), for instance, excessive membrane swelling may cause anode flooding, impaired gas transport, and mechanical failure [2, 17]. Accordingly, a central challenge in AEM research lies in mitigating water uptake and dimensional swelling while maintaining high ionic conductivity.

**SCHEME 1:**
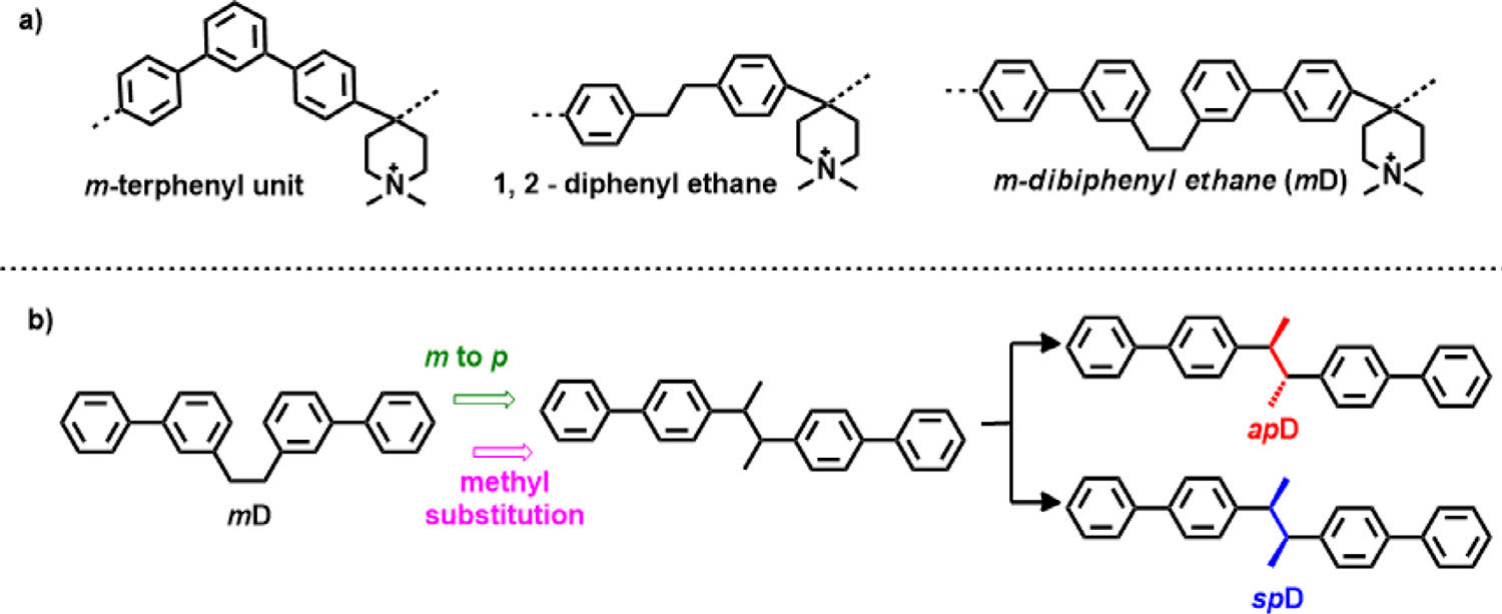
Structural motifs and monomer designs for poly(arylene piperidinium): (a) representative backbone units used to introduce conformational flexibility without any hetero‐atoms, [31, 35, 36] (b) transformation of the *m*D unit into the p‐connected, benzylic‐methyl‐substituted unit, giving rise to the diastereomeric *ap*D and *sp*D monomers.

Controlling polymer chain flexibility has emerged as an effective strategy for modulating the structure–property relationships of AEMs [18, 19, 20, 21, 22, 23, 24, 25, 26, 27, 28]. Polymer flexibility usually increases the degree of hydration and the local mobility of both the cationic groups and the polymer backbone, thereby promoting hydroxide transport through the formation of ion‐conducting domains, while also improving alkaline stability [16, 17, 18, 19, 20, 21, 22, 23, 24, 25, 26, 27, 28, 29, 30]. Flexibility can be introduced into either the polymer backbone or through side chains tethering the cations to the polymer. Owing to the broad availability of flexible building blocks and the many synthetic possibilities, side‐chain engineering has led to various successful AEMs, including the commercially available Orion membrane [21, 22, 24]. In contrast, synthetic strategies that introduce backbone flexibility, while avoiding heteroatom linkages, remain limited [31, 32, 33, 34, 35, 36]. Angled arene units [31, 32, 33] (e.g., *m*‐terphenyl) and short alkyl bridges [34, 35] (e.g., bibenzyl) represent two of the most widely adopted motifs for introducing conformational freedom into otherwise rigid aromatic polymer backbones (Scheme 1a).

To explore the effect of increasing backbone flexibility on AEM properties, we recently developed a monomer (*m*D) which integrates both angled arene units and a short alkyl segment within a single repeat unit (Scheme 1a) [36]. This design was intended to promote ion‐domain organization at reduced IECs by introducing substantial conformational freedom to the aromatic polymer backbone. However, despite the improved ion‐domain organization, revealed by small‐angle X‐ray scattering, the *m*D‐based membranes exhibited markedly increased water uptake, even at reduced IEC. The resulting excessive swelling substantially limits their applicability in electrochemical systems with stringent water‐management requirements. These observations suggest that simply increasing backbone flexibility to benefit ion transport may simultaneously lead to uncontrolled hydration.

Motivated by this limitation, we have now designed a new arene monomer with the aim to locally increase the conformational freedom at a defined molecular motif, rather than broadly increasing the overall backbone flexibility. This was achieved by replacing the *meta*‐linked arene structure of *m*D with a *para*‐linked analogue, while also introducing two benzylic methyl substituents to restrict the alkyl bond rotation. Such a design is intended to confine conformational freedom to a specific local structural element, without substantially altering the overall backbone architecture.

As a direct consequence of this design, the resulting monomer is obtained as two diastereomers, denoted as *ap*D [*anti*‐*p*‐di(biphenyl)dimethylethane] and *sp*D [*syn*‐*p*‐di(biphenyl)dimethylethane] (Scheme 1b). Although the stereogenic center is not located in the immediate vicinity of the ionic groups, previous studies have shown that subtle diastereomeric differences can give rise to pronounced variations in polymer properties [37]. Zhao and co‐workers have reported that diastereomeric polyesters derived from bisphenols bearing vicinal trifluoromethyl substituents exhibited markedly different physical properties, despite having identical chemical compositions, which was attributed to their distinct solid‐state packing and crystallinity [37]. This finding highlights the capability of highly localized stereochemical perturbations to impose distinct chain geometries—analogous to the *E*/*Z* configurations in unsaturated polymers—and thereby influence macroscopic polymer properties. Inspired by this example, we hypothesized that analogous diastereomeric effects may be exploited to modulate the ion‐transporting microenvironment in AEMs, namely the local physicochemical surroundings of the ionic groups, including their hydration characteristics and chain packing environment arising from the backbone conformation.

Accordingly, *ap*D‐ and *sp*D‐enriched monomers were synthesized and copolymerized with *p*‐terphenyl to afford a family of ether‐free poly(arylene piperidinium) AEMs. Comparison of the resulting *anti*‐ and *syn*‐enriched membranes with the same compositions enables systematic investigation of how subtle stereochemical variations influence membrane hydration, ion transport, alkaline stability, and mechanical properties. Selected membranes were further evaluated in AEMWE cells to assess the practical implications and benefits of these molecular‐level effects under operating conditions.

## 2 Results and Discussion

### 2.1 Monomer Design and Material Synthesis

To gain qualitative insight into the local conformational preferences of the diastereomeric units, dihedral angle scans about the backbone linkage adjacent to the stereogenic center were performed using density functional theory (DFT) calculations on *ap*D and *sp*D model compounds (Figure S5). Although based on simplified molecular fragments in the absence of a condensed‐phase environment, the calculations revealed distinct conformational tendencies arising from the different stereochemical arrangements. In particular, the *syn* diastereomer (*sp*D) more readily adopted kinked geometries with relatively lower energetic penalties, whereas the *anti* diastereomer (*ap*D) favored more extended conformations.

**SCHEME 2:**
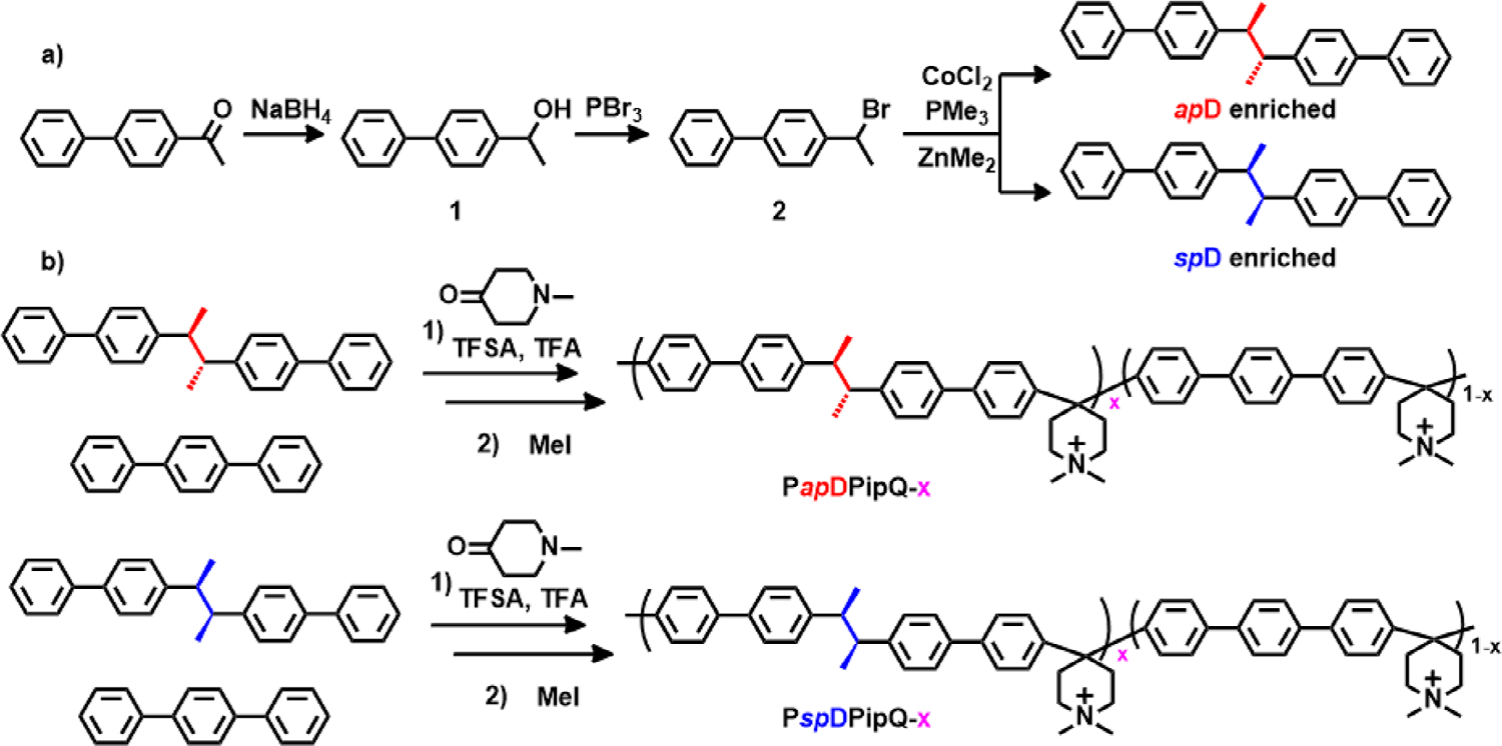
(a) Synthetic strategies for the monomers via cobalt‐catalyzed reductive homocoupling reaction, and (b) Polymer synthesis via polyhydroxyalkylation, *x* indicating the mol% of *ap*D or *sp*D units.

We next synthesized the diastereomeric monomers and prepared the corresponding poly(arylene piperidinium)s (Scheme 2). A crucial advantage of AEM‐based electrochemical devices is the possibility to employ non‐precious metal electrocatalysts under alkaline conditions [1, 2]. In this context, all three steps of the monomer synthesis were designed to avoid the use of noble‐metal catalysts in order to meet the sustainability and cost considerations associated with AEM technologies. The final key bond‐forming step was achieved through a cobalt‐catalyzed reductive homocoupling reaction, following the protocol previously developed for the *m*D monomer [37, 38].

**FIGURE 1:**
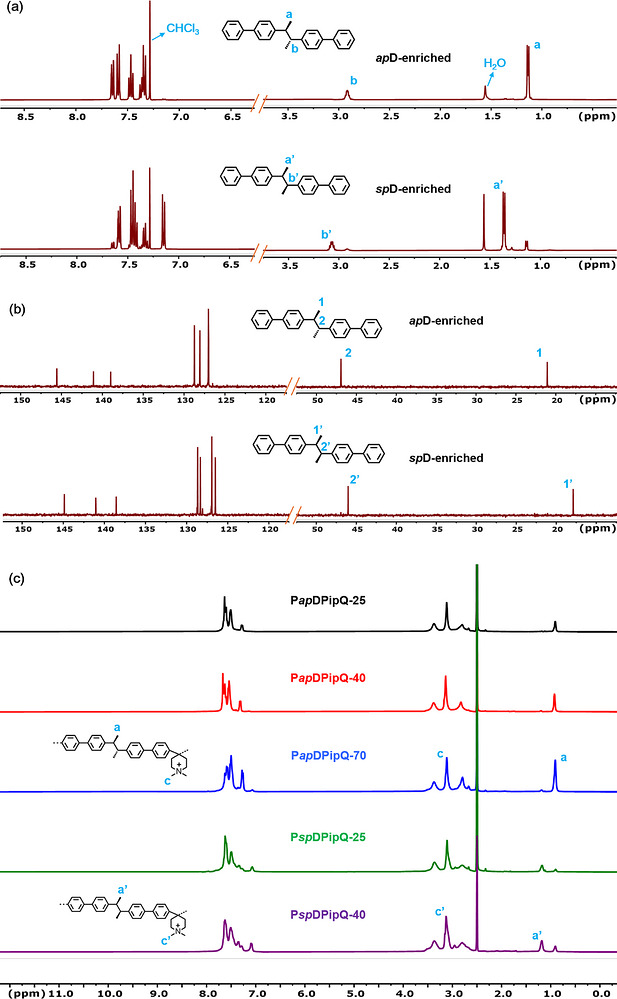
(a) ^1^H and (b) ^13^C NMR spectra of the *ap*D‐ and *sp*D‐ enriched monomer samples recorded in chloroform‐*d*, and (c) ^1^H NMR spectra of the samples in the P*ap*DPipQ‐*x* and P*sp*DPipQ‐*x* series recorded using DMSO‐*d*_6_ solutions. Trifluoroacetic acid (TFA) was added to shift the water signal.

The cobalt‐catalyzed reductive homocoupling proceeds via a single‐electron‐transfer pathway, in which the C(sp^3^)–C(sp^3^) bond is formed through radical recombination [38, 39]. Under the reaction conditions employed here, no obvious diastereoselectivity toward either the *syn* or *anti* diastereomer (*sp*D or *ap*D) was observed. The *sp*D monomer was obtained as a racemic mixture of enantiomers (*R,R*) and (*S,S*); for clarity, only one enantiomer is depicted in Scheme 2. Owing to the markedly lower solubility of *ap*D in common organic solvents, it readily precipitated during work‐up and was isolated by simple filtration, affording an *ap*D‐enriched monomer mixture with a diastereomeric purity of approximately 95%. In contrast, although *sp*D exhibited good solubility in heptane and dichloromethane, a pronounced tailing of *ap*D during column chromatography rendered complete separation difficult, resulting in an *sp*D‐enriched monomer mixture with a diastereomeric purity of approximately 84% (Figure 1a).

The chemical structures and contents of the *ap*D‐ and *sp*D‐enriched monomers were confirmed by ^1^H NMR spectroscopy (Figure 1a,b). The signals of the methyl protons of *ap*D and *sp*D were observed at 1.13 and 1.36 ppm, respectively, while the corresponding benzylic protons were observed at 2.91 and 3.07 ppm, respectively. The diastereomeric enrichments were determined by integration of these characteristic ^1^H NMR signals. Notably, the two diastereomeric‐enriched monomer samples already exhibited distinctly different physical properties. The *ap*D‐enriched monomer sample displayed a markedly lower solubility and a substantially higher melting temperature (260–263°C) than the *sp*D‐enriched counterpart (138–141°C). Differential scanning calorimetry (DSC) further confirmed melting transitions consistent with the measured melting points (Figure S6).

The *ap*D‐ and *sp*D‐enriched monomers were subsequently used in copolymerizations with *p*‐terphenyl to afford a series of poly(arylene piperidinium) copolymers denoted as P*ap*DPipQ‐*x* and P*sp*DPipQ‐*x*, where *x* indicates the mol% of *ap*D‐ or *sp*D‐enriched units in the polymer (Scheme 2b). Owing to the lower solubility of the *ap*D‐enriched monomer sample, the incorporation of this monomer in the resulting copolymers was lower than in the corresponding feed ratio. In contrast, the incorporation of the *sp*D‐enriched monomer closely matched the feed ratio under otherwise identical polymerization conditions. To enable a direct comparison between *anti*‐ and *syn*‐based AEMs, the feed ratios of the *sp*D‐enriched monomer sample were adjusted to match the experimentally determined incorporation ratios of *ap*D. As a result, a set of copolymers with very similar monomer compositions was obtained (Figure 1c), namely P*ap*DPipQ‐25, P*ap*DPipQ‐40, P*ap*DPipQ‐70, P*sp*DPipQ‐25, and P*sp*DPipQ‐40. A P*sp*DPipQ‐70 sample was prepared but did not form free‐standing membranes. This sample showed a lower intrinsic viscosity (0.33 dL g^−1^) than the other polymers in the present series. In addition, a gradual decrease in the diastereomeric purity of the *sp*D component inserted in the polymer was observed during copolymerizations, suggesting a lower effective reactivity of *sp*D compared with *ap*D. Specifically, the *sp*D content decreased from 84% in the *sp*D‐enriched monomer to 79% in P*sp*DPipQ‐25, and further to 75% in P*sp*DPipQ‐40. The diastereomeric purity of the *ap*D component was approximately 95% both in the mixture before the polymerizations and in the samples of the P*ap*DPipQ‐*x* series. Because the molecular weight is an important parameter, governing the physical and transport properties of AEMs, the intrinsic viscosities of the polymers were measured at 25°C in DMSO containing 0.1 M LiBr using an Ubbelohde viscometer. All five copolymers exhibited intrinsic viscosities in the narrow range 0.41–0.48 dL g^−^^1^, indicating comparable molecular weights across the series.

Freestanding membranes with a thickness of approximately 60 µm were cast from DMSO solutions. The membranes were transparent and mechanically robust, and were subsequently converted to the Br^−^ form. The ion‐exchange capacity (IEC) was determined by Mohr titrations (Table 1), and the *ap*D and *sp*D contents of the copolymers were estimated based on the NMR and titration results (Table S1).

**TABLE 1:** Properties of the P*ap*DPipQ‐*x* and P*sp*DPipQ‐*x* AEMs.

AEMs	IEC ^a^ (mequiv. g^−1^)	Water uptake ^b^ (%)		2 M KOH ^b^ uptake (%)		OH^−^ conductivity in water (mS cm^−1^)		*T*_d,95_ ^c^ (°C)	[η]^c^ (dL g^−1^)
		20°C	80°C	20°C	80°C	20°C	80°C		
P*ap*DPipQ‐25	2.57	65	167	52	71	85	188	268	0.48
P*ap*DPipQ‐40	2.46	58	148	50	68	72	160	272	0.46
P*ap*DPipQ‐70	2.23	43	93	48	67	79	180	272	0.46
P*sp*DPipQ‐25	2.56	74	255	52	97	91	198	273	0.42
P*sp*DPipQ‐40	2.43	60	163	45	72	77	165	295	0.41

^a^ Titrated IEC, measured in the Br^–^ form, then calculated and presented in the OH^–^ form;

^b^ Measured in the OH^–^ form;

^c^ Measured in the Br^–^ form.

### 2.2 Mechanical Properties

**FIGURE 2:**
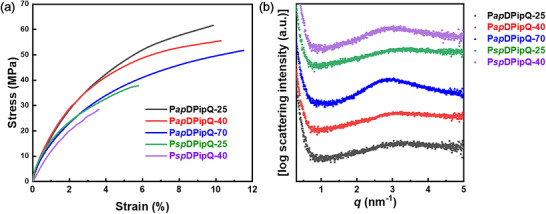
(a) Stress‐strain curves of dry AEMs measured at a constant 0.5 N min^−1^ ramping force, and (b) SAXS profiles of dry AEMs in the Br^–^ form.

Mechanical robustness of the membrane is a prerequisite for reliable assembly and operation of AEM‐based devices. The mechanical properties of the membranes in the P*ap*DPipQ‐*x* and P*sp*DPipQ‐*x* series were evaluated by tensile testing under a constant 0.5 N min^−1^ ramping force. As shown in Figure 2a, increasing *ap*D contents led to a gradual decrease of the stress at break, accompanied by an increase in the strain at break, indicating an increase in ductility. In contrast, an increasing *sp*D content resulted in a simultaneous decrease of both the stress and strain at break, suggesting increased brittleness. This trend was also consistent with the unsuccessful preparation of a free‐standing P*sp*DPipQ‐70 membrane, although the lower intrinsic viscosity of this sample may also have contributed to the poor film‐forming ability of this sample. Young's modulus was determined from the initial linear region of the stress–strain curves (0–2% strain). The P*ap*DPipQ‐*x* series consistently exhibited higher moduli than the corresponding P*sp*DPipQ‐*x* membranes. Hence the values were 1.39, 1.37, and 1.05 GPa for P*ap*DPipQ‐25, ‐40, and ‐70, respectively, compared to 1.13, and 1.01 GPa for P*sp*DPipQ‐25 and ‐40, respectively. In view of the distinctly different local conformational preferences revealed by the dihedral angle analysis, the lower modulus and inferior mechanical robustness of the AEMs in the P*sp*DPipQ‐*x* series were tentatively attributed to their tendency to adopt more locally kinked backbone conformations, which may lead to less efficient chain packing, reduced entanglement density, and weakened interchain interactions.

### 2.3 Morphology

Increasing the hydrophobic content of an AEM is often associated with competing effects on ion‐domain formation and connectivity [40]. In this work, replacing rigid *p*‐terphenyl segments with flexible *ap*D or *sp*D units increased the local conformational freedom of the backbone. This structural feature facilitates the development of ion‐conducting domains even as the IEC decreases. As shown in Figure 2b, the intensity of the ionomer peak in the SAXS profiles of the P*ap*DPipQ‐*x* series increased systematically with increasing *ap*D content (from 25% to 70%), despite the simultaneous decrease in IEC from 2.57 to 2.24 mequiv. g^−^^1^. P*ap*DPipQ‐70 exhibited the strongest scattering intensity, while the characteristic domain distance remained essentially unchanged at approximately 2.1 nm, indicating a similar average domain spacing but enhanced ion‐domain organization. A similar trend was observed for the P*sp*DPipQ‐*x* series; P*sp*DPipQ‐40 displayed a slightly higher scattering intensity than P*ap*DPipQ‐40 at the same composition. These trends were consistent with the AFM phase images, which showed enhanced nanoscale phase contrast with increasing *ap*D and *sp*D content, respectively, indicating more distinct phase separation (Figure S7). Together, the SAXS and AFM analyses support our design rationale that locally flexible units facilitate the formation of ion‐conducting domains at reduced IEC.

### 2.4 Aqueous Uptake and Swelling

**FIGURE 3:**
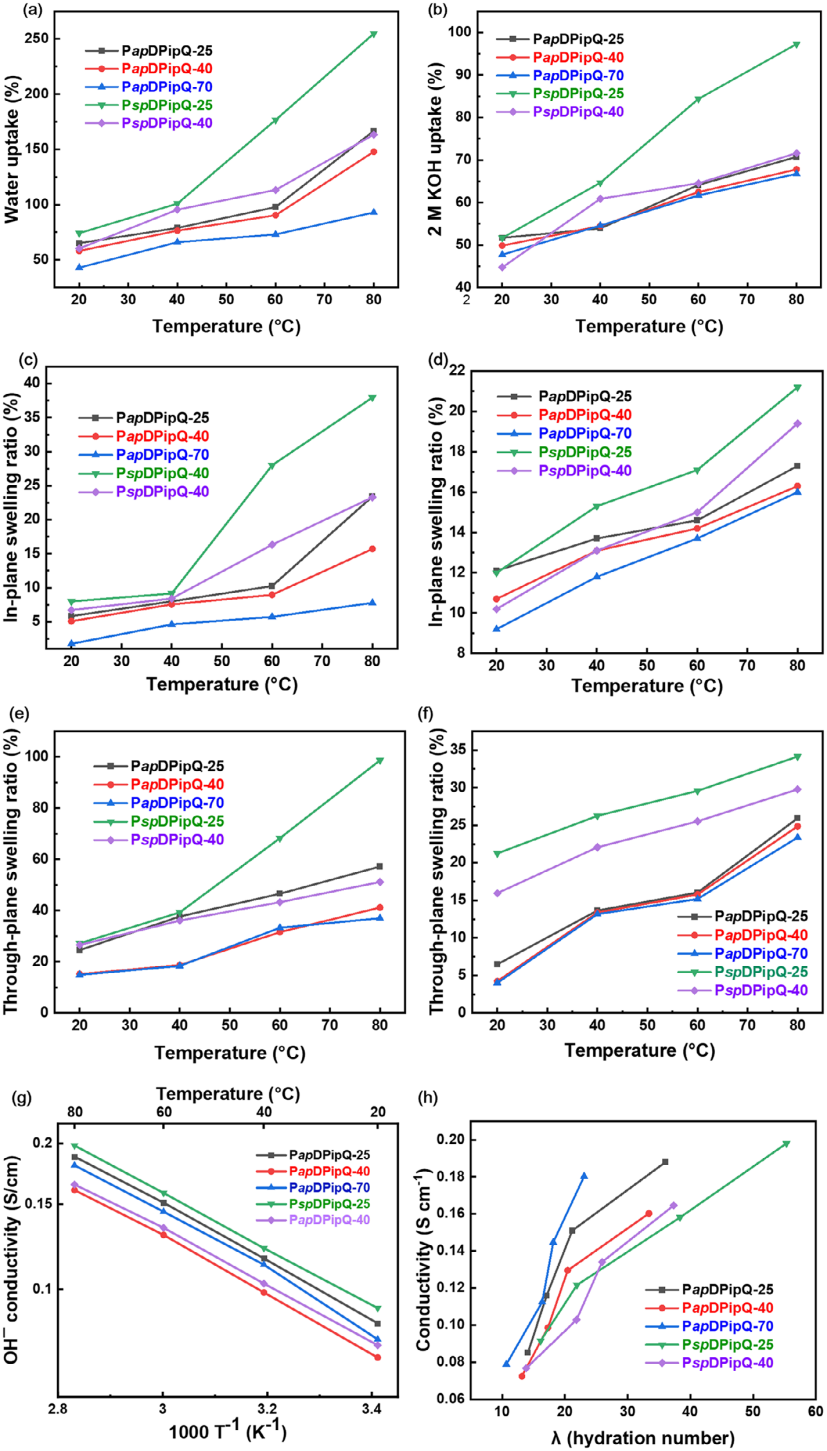
Water uptake, swelling and ionic conductivity data of the AEMs in the P*ap*DPipQ‐*x* and P*sp*DPipQ‐*x* series between 20 and 80°C: uptake of water (a) and 2 M KOH (aq.) solution (b), in‐plane swelling ratio in water (c) and in 2 M KOH (aq.) solution (d), through‐plane swelling ratio in water (e) and in 2 M KOH (aq.) solution (f), as well as the OH^–^ conductivity of the AEMs in the fully hydrated (immersed) state as a function of T^−1^ (g) and the hydration number λ (h).

Water uptake is a critical parameter governing both the dimensional stability, ionic conductivity, and the practical operation of AEM‐based devices. The water uptake and swelling behavior of the P*ap*DPipQ‐*x* and P*sp*DPipQ‐*x* membrane series were examined gravimetrically (Figure 3a). In both series, the water uptake decreased monotonically with decreasing IEC, corresponding to increasing *ap*D and *sp*D contents. At comparable IEC values, the P*sp*DPipQ‐*x* membranes consistently exhibited significantly higher water uptake than their P*ap*DPipQ‐*x* counterparts. The in‐ and through‐plane swelling ratios generally followed the same trend as the water uptake (Figure 3c,e), with the exception that the through‐plane swelling ratios of P*ap*DPipQ‐40 and P*ap*DPipQ‐70 were nearly identical. Although both *ap*D and *sp*D introduce localized flexibility into the polymer backbone, and do not substantially alter the characteristic length scale of the phase separation, the distinct difference between the two different diastereomer‐enriched membrane series indicates that the *anti*‐ and *syn*‐enriched moieties differed in their influence on membrane hydration. The higher water uptake of the P*sp*DPipQ‐*x* membranes was tentatively ascribed to the distinct local conformational preferences of the *sp*D units. This assumption was consistent with the lower Young's moduli of the P*sp*DPipQ‐*x* membranes, suggesting a less stiff polymer matrix that may provide weaker elastic resistance against hydration‐induced swelling and water uptake. For example, P*sp*DPipQ‐25 exhibited the highest water uptake among the membranes investigated, exceeding 250% at 80°C (99% through‐plane swelling), whereas P*ap*DPipQ‐25 showed a water uptake of 166% at 80°C (57% through‐plane swelling). Furthermore, P*ap*DPipQ‐70 displayed the lowest water uptake, remaining below 100% at 80°C (37% through‐plane swelling). In sharp contrast, the previously reported P*m*DPipQ‐*x* series of AEMs based on the *m*D monomer displayed an unusual increase in water uptake with decreasing IEC [28]. Notably, P*m*DPipQ‐50, with a similar IEC and a higher intrinsic viscosity (0.82 dL g^−1^), exhibited a water uptake exceeding 500% at 80°C. This comparison highlights the markedly suppressed hydration of the present *ap*D‐ and *sp*D‐based membranes relative to the previously studied *m*D‐based AEMs, demonstrating that the introduction of local chain flexibility through the *ap*D‐ and *sp*D‐units effectively mitigated the excessive swelling allowed by the more globally flexible *m*D‐containing backbone.

Similar to the water‐uptake data, the uptake of 2 M KOH (aq.) solution and the corresponding swelling ratios decreased monotonically with decreasing IEC, and the P*sp*DPipQ‐*x* series consistently exhibited a higher uptake than the corresponding P*ap*DPipQ‐*x* membranes at comparable IEC values. For example, the uptake of 2 M KOH (aq.) by the P*sp*DPipQ‐25 and P*ap*DPipQ‐25 AEMs reached 97 and 71%, respectively, at 80°C. Although the overall trend closely followed that observed for the water uptake, the absolute uptake of the KOH solution was substantially lower than the water uptake. These observations were consistent with the structural differences between the two diastereomeric series.

### 2.5 OH^−^ Conductivity

Ion conductivity is a key performance metric for AEMs and is governed by multiple factors, including temperature, ion‐exchange capacity, hydration level, and membrane morphology. Because the incorporation of *ap*D or *sp*D units simultaneously suppressed water uptake and promoted the formation of distinct ion‐conducting domains, the hydroxide conductivity of the P*ap*DPipQ‐*x* and P*sp*DPipQ‐*x* membranes was studied in detail to assess the combined influence of hydration and morphology on ion transport (Figure 3g).

Consistent with the highest water uptake, P*sp*DPipQ‐25 exhibited the highest hydroxide conductivity, very close to 200 mS cm^−^^1^. At comparable IEC values, the P*sp*DPipQ‐*x* membranes also consistently showed higher hydroxide conductivity than their P*ap*DPipQ‐*x* counterparts. However, the conductivity difference between the P*ap*DPipQ‐*x* and P*sp*DPipQ‐*x* series was relatively modest compared with the variation observed within each series. This observation suggested that, within the present compositional window, the IEC remains the dominant factor governing hydroxide conductivity.

Notably, the OH^−^ conductivity within the P*ap*DPipQ‐*x* series did not vary monotonically with the water uptake and *ap*D content. In particular, P*ap*DPipQ‐70 showed a higher OH^−^ conductivity than P*ap*DPipQ‐40. The former AEM possessed the lowest IEC and the lowest water uptake but showed the strongest ionomer peak intensity by SAXS, indicative of a more developed and organized phase‐separated morphology. Hence, the OH^−^ conductivity of P*ap*DPipQ‐70 reached 180 mS cm^−^^1^ at a water uptake below 100%. These results indicated that P*ap*DPipQ‐70 achieved a markedly higher hydration efficiency for ion transport compared to the membranes investigated in this work (Figure 3h). Moreover, this behavior demonstrated that the present structural design enabled efficient OH^−^ transport at a substantially reduced hydration level, which is highly desirable for electrochemical applications requiring stringent water management.

### 2.6 Thermal and Alkaline Stability

The thermal stability of the P*ap*DPipQ‐*x* and P*sp*DPipQ‐*x* membranes was evaluated by thermogravimetric analysis (TGA) under a nitrogen atmosphere (Figure S8). All membranes were found to degrade in a multistep thermal decomposition process, and the temperatures at 5% weight loss (*T*_d,95_) were all above 260°C, indicating that the present structure design did not compromise the thermal robustness of the piperidinium‐based membranes. P*sp*DPipQ‐40 showed the highest *T*_d,95_ at approximately 295°C. This slightly higher thermal stability may be related to the high content of the more locally flexible *sp*D units, in line with previous observations that a higher backbone flexibility may improve the thermal stability of poly(arylene piperidinium) AEMs [18, 36].

**FIGURE 4:**
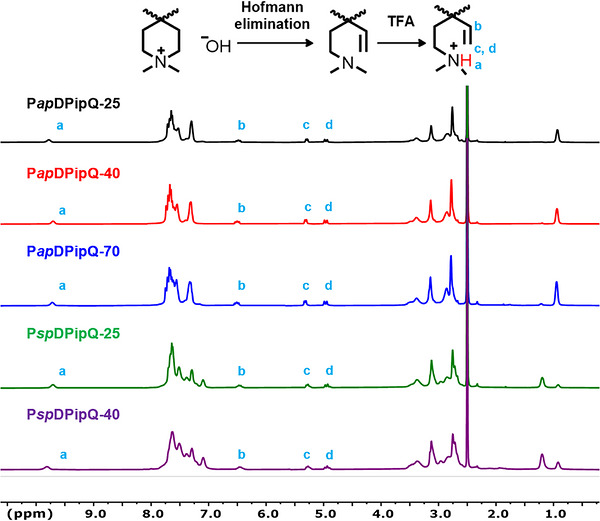
^1^H NMR spectra of AEM samples after storage in 5 M KOH (aq.) solutions at 90°C for 480 h, displaying small signals (a–d) emerging from the degradation products formed by Hofmann β‐elimination. TFA was added to protonate the tertiary amine groups originating from degradation reactions and to shift the water signal.

To investigate whether the diastereomeric units affect the alkaline stability of the resulting AEMs, membrane samples were stored in 5 M KOH (aq) at 90°C for 480 h prior to ^1^H NMR analysis to identify structural changes. An accelerated alkaline test was applied to clarify potential differences between the two diastereomeric AEM series. After treatment in KOH solution, the ^1^H NMR spectra of both P*ap*DPipQ‐*x* and P*sp*DPipQ‐*x* membranes exhibited signals for degradation products at 9.7, 6.5, 5.3, and 5.0 ppm with an integral ratio of 1:1:1:1, indicating ionic loss associated with Hofmann β‐elimination (Figure 4). This observation is consistent with the previously reported P*m*DPipQ‐*x* series, in which ionic loss was dominated by Hofmann β‐elimination [28]. Among these results, P*ap*DPipQ‐25, P*ap*DPipQ‐40, and P*ap*DPipQ‐70 showed approximately 37%‐38% ionic loss, as estimated from integrals of the NMR signals. In comparison, P*sp*DPipQ‐25 and P*sp*DPipQ‐40 exhibited approximately 31% ionic loss, indicating improved alkaline stability relative to the *ap*D‐enriched counterparts.

Notably, both P*ap*DPipQ‐*x* and P*sp*DPipQ‐*x* membranes exhibited lower ionic loss compared to AEMs based on a backbone based on purely *p*‐terphenyl units (42% ionic loss for PTPipQ100) [18], which is consistent with previous observations that increasing backbone flexibility can enhance alkaline stability [41]. In contrast, the more subtle difference between the *ap*D‐ and *sp*D‐enriched membranes, which share the same chemical structure but differ in local stereochemistry, is less likely to arise from global backbone flexibility alone. Instead, this difference may be associated with variations in local conformational constraints, chain packing, and hydration environment around the cationic groups.

### 2.7 Water Electrolysis Evaluation

**FIGURE 5:**
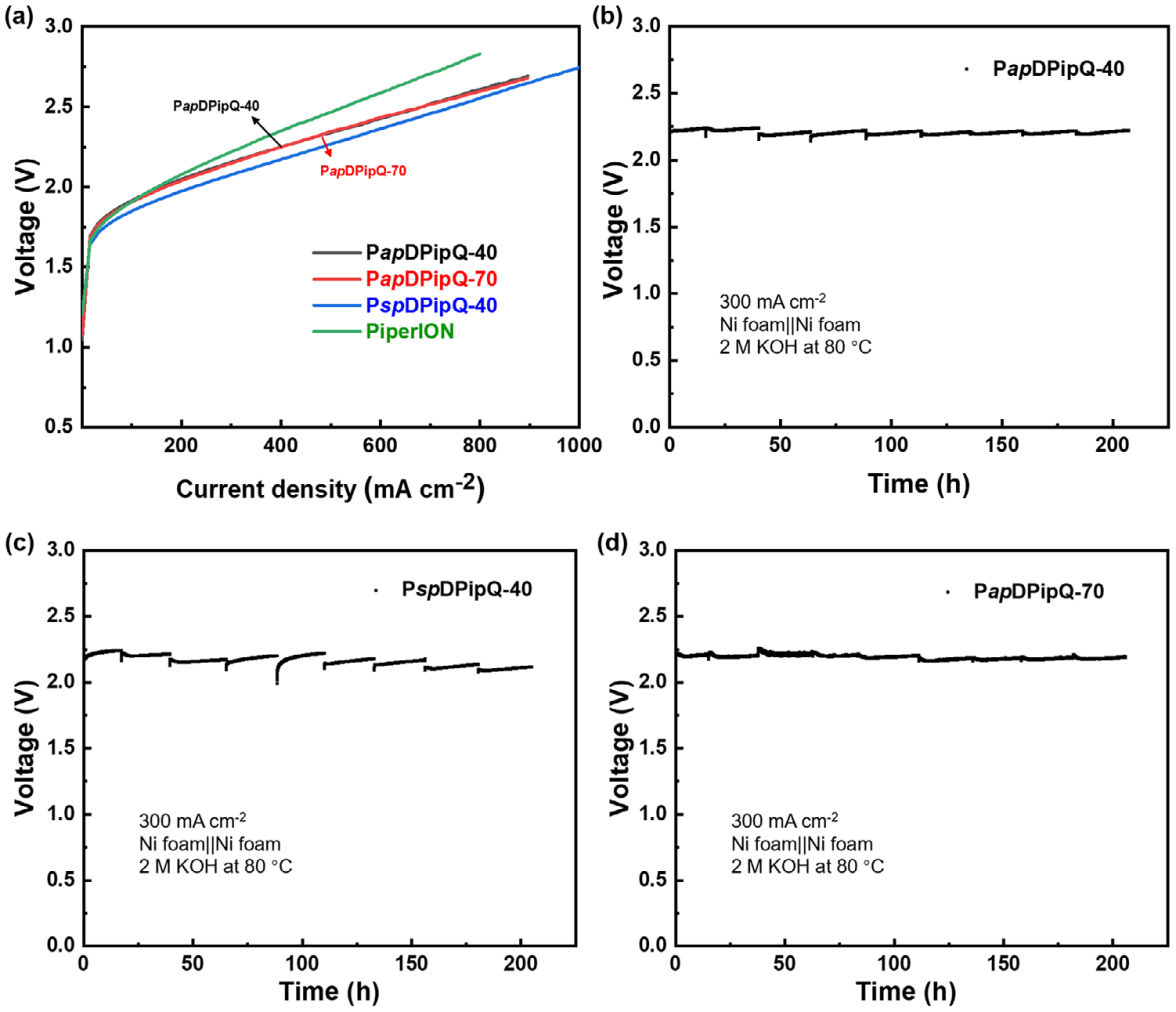
a) Polarization curves of P*ap*DPipQ‐40, P*ap*DPipQ‐70, P*sp*DPipQ‐40 and PiperION 60 membrane recorded at 80°C with a feed of 2 M KOH AEMWE, and operational stability of the cells assembled with b) P*ap*DPipQ‐40, c) P*sp*DPipQ‐40 and d) P*ap*DPipQ‐70 during 200 h at 300 mA and 80°C in 2 M KOH solution.

To investigate how the macromolecular design and subtle stereochemical differences influence the electrochemical performance, three representative membranes—P*ap*DPipQ‐40, P*sp*DPipQ‐40, and P*ap*DPipQ‐70—were selected for an initial AEMWE evaluation. Comparison of the performance of P*ap*DPipQ‐40 and P*sp*DPipQ‐40 enabled a direct assessment of the stereochemical effects at comparable IEC, whereas P*ap*DPipQ‐40 and P*ap*DPipQ‐70 allowed an evaluation of the influence of the *ap*D content on membrane performance. PiperION 60 was included as a reference material to contextualize the device performance of the present membranes. Membranes with a thickness of ∼60 µm were assembled in a single‐cell setup with an active area of 6.25 cm^2^, using plain uncatalyzed nickel foam for both electrodes and 2 M KOH (aq). solution as electrolyte. The resulting polarization curves recorded at 80°C are shown in Figure 5a. At low current densities, the polarization curves largely overlapped, indicating comparable baseline losses and good reproducibility of the cell assembly.

At a voltage of 2.50 V, cells assembled with P*ap*DPipQ‐40, P*ap*DPipQ‐70, and P*sp*DPipQ‐40 reached current densities of 670, 673, and 747 mA cm^−^^2^, respectively, with P*sp*DPipQ‐40 exhibiting the highest current density among the three membranes. Notably, all three membranes outperformed the commercial PiperION reference membrane operating with the same cell and uncatalyzed nickel foam electrodes (538 mA cm^−^^2^). The relative performance trend of the P*ap*DPipQ‐40, P*ap*DPipQ‐70, and P*sp*DPipQ‐40 membranes was consistent with the uptake of 2 M KOH (aq.) solution, suggesting that membrane hydration under alkaline conditions played an important role for the observed AEMWE performance. The corresponding EIS spectra showed comparable charge‐transfer resistances (2.7–2.8 Ω cm^2^) for all cells, while the cells assembled with the present membranes exhibited lower ohmic resistances (0.6 Ω cm^2^) than that with PiperION 60 (0.9 Ω cm^2^) (Figure S10).

It should be noted that PiperION is a membrane designed for robust operation of a broad range of different conditions, while the present membranes were primarily developed to study structure–property relationships. Moreover, all measurements were conducted using simple nickel foam electrodes without the use of advanced catalysts, leaving room for further performance improvements through electrode optimization. Nevertheless, the comparable, and even higher, current densities obtained under the same testing conditions demonstrated that the present membranes can deliver high‐level electrochemical performance in AEMWE and do not merely represent conceptual advances at the materials level.

**FIGURE 6:**
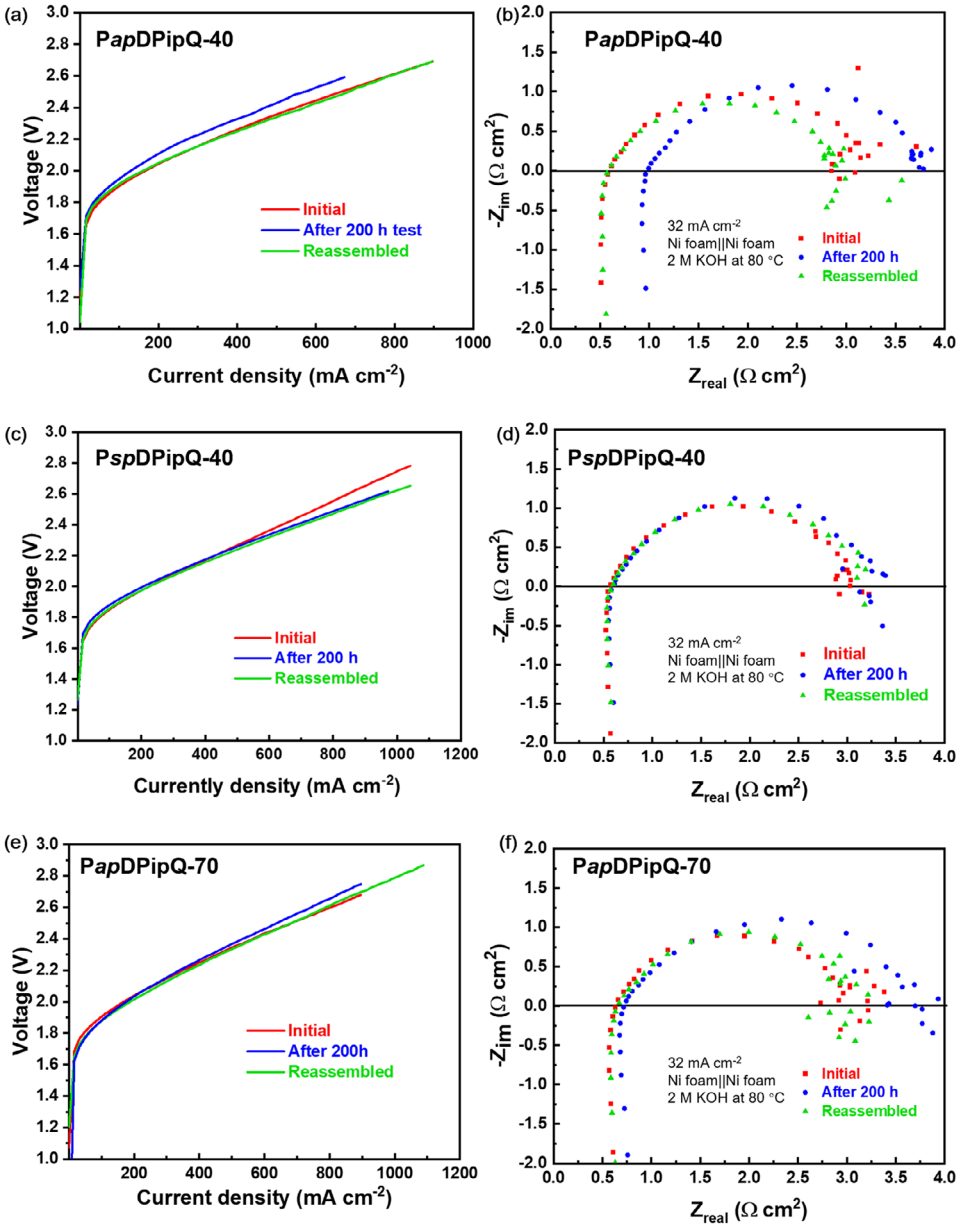
Polarization curves of (a) P*ap*DPipQ‐40, (c) P*sp*DPipQ‐40, and (e) P*ap*DPipQ‐70 measured before and after 200 h of AEMWE operation in 2 M KOH at 80°C, as well as after reassembly of the cell with fresh nickel foam electrodes. Corresponding galvanostatic electrochemical impedance spectra (GEIS) of (b) P*ap*DPipQ‐40, (d) P*sp*DPipQ‐40, and (f) P*ap*DPipQ‐70 recorded before and after 200 h of AEMWE operation in 2 M KOH at 80°C, and after reassembly with fresh nickel foam electrodes (measured at 32 mA cm^−^^2^).

Durability tests were performed under galvanostatic conditions at 300 mA cm^−^^2^ in 2 M KOH (aq.) solution at 80°C to further evaluate the practical applicability of the present membranes during AEMWE operation (Figure 5b–d). The cell voltage required to maintain the applied current density remained largely stable during 200 h of continuous operation. However, the polarization curves recorded after 200 h of AEMWE operation (Figure 6a,e) revealed a moderate performance loss for the cells with P*ap*DPipQ‐40 and P*ap*DPipQ‐70, respectively, as evidenced by the reduced current densities reached at identical cell voltages compared with the original values. In contrast, no performance loss was detected for the cell with P*sp*DPipQ‐40 in the corresponding polarization curves after 200 h of operation (Figure 6c).

Consistently, the galvanostatic electrochemical impedance spectroscopy (GEIS) measurements showed an increase in the high‐frequency resistance (HFR) for P*ap*DPipQ‐40 and P*ap*DPipQ‐70 after 200 h of operation, while no HFR increase was observed for P*sp*DPipQ‐40, indicating that the resistance buildup is specific to the P*ap*DPipQ‐based cells. After disassembling the cells, a pronounced darkening of the nickel foam anode, as well as the corresponding current collectors, was observed for the cells assembled with the P*ap*DPipQ‐40 and P*ap*DPipQ‐70 membranes (Figure S9). This observation is consistent with the formation of oxidized nickel phases under oxygen evolution reaction conditions during prolonged alkaline operation at high voltages, as previously reported for nickel‐based electrodes [42, 43]. In contrast, no visible darkening was observed for the electrodes and current collectors in the cell with the P*sp*DPipQ‐40 membrane. This difference may further reflect the distinct cell behavior of the diastereomer‐derived membranes during AEMWE operation.

After the initial evaluation, the cells were reassembled using fresh nickel foam electrodes without exchanging the AEMs. As shown in Figure 6a,e, the performance loss observed after 200 h of operation was largely recovered after the reassembly. Consistently, the GEIS results indicated that the increase in HFR occurring during the 200 h operation was also largely reversed after reassembly (Figure 6b,f). Combined with the fact that NMR results confirmed that the chemical structures of the AEMs remained unchanged after 200 h of operation (Figure S12), these observations suggested that the performance losses were primarily associated with changes at the nickel foam electrodes rather than the membranes.

## 3 Conclusions

In the present work, we designed and synthesized two series of diastereomeric anion exchange membranes based on a diastereomeric monomer platform that enables control over localized chain flexibility within an otherwise rigid backbone and demonstrated that this design provides an effective strategy to promote ion‐domain development from excessive swelling. Building on our previous *m*D moiety, the present locally flexible monomer design preserves the ability to promote ion‐conducting domain formation at reduced ion‐exchange capacities, while simultaneously suppressing the uncontrolled water uptake observed for the *m*D‐based membranes. Furthermore, the incorporation of *ap*D‐ and *sp*D‐units constituted a minimal and well‐defined structural variation, enabling a systematic assessment of how subtle stereochemical variations regulate AEM properties. By comparing the two diastereomeric AEM series, we showed that *syn*‐enriched membranes consistently exhibited higher water uptake than their *anti*‐counterparts at comparable IEC, which was accompanied by higher hydroxide conductivity and improved AEMWE performance. In addition, AEMWE cells equipped with *syn*‐enriched AEMs displayed superior operational stability compared with those employing *anti*‐enriched AEMs. In contrast, *anti‐*enriched AEMs exhibited improved mechanical robustness and higher hydration efficiency for ion transport. These results indicate that such minimal stereochemical variations are associated with systematic differences in macroscopic membrane properties, consistent with subtle differences in local backbone geometry. This implies that the present strategy may be extended to other AEM systems in which the cationic groups are incorporated into, or located close to, the polymer backbone. Moreover, the present methyl‐substituted *ap*D/*sp*D pair represents only one example of diastereomeric backbone modulation. Based on the synthetic route described in this work, other functional groups may be introduced via the synthesis of corresponding functionalized benzyl bromide precursors, thereby expanding the molecular toolbox for tuning local backbone conformation, hydration behavior, and ion transport in AEMs. Overall, the diastereomeric backbone design provides a unique molecular platform that enables structure–property correlations based on subtle local conformational variations under well‐controlled structural parameters, thereby facilitating a rational design of high‐performance ion‐conducting polymers.

## Author Contributions

**Triet Nguyen Dai Luong**: methodology, formal analysis. **Xuchen Lyu**: investigation, formal analysis. **Patric Jannasch**: funding acquisition, resources, supervision, writing – review and editing. **Si Chen**: conceptualization, formal analysis, methodology, investigation, validation, writing – original draft.

## Funding

Swedish Foundation for Strategic Research (SSF) through the “PUSH” project (grant ARC19‐0026), the Swedish Energy Agency (grants 50519‐1, 45057‐1, 37806‐3, and 45515‐3), the Swedish Research Council (grant 2023–04525), and the Royal Physiographic Society in Lund.

## Conflicts of Interest

The author declares no conflicts of interest.

## Supporting information




**Supporting File**: advs76588‐sup‐0001‐SuppMat.pdf.

## Data Availability

Experimental details, DFT calculations, additional characterization (AFM, TGA, and NMR), and additional electrochemical data are provided in the Supporting Information. The authors have cited additional references within the Supporting Information [44].
