# Supplementary material for: Diastereomeric Monomers Enable Anion‐Exchange Membranes With Controlled Local Polymer Backbone Flexibility and High Conductivity
Source: Adv Sci (Weinh). 2026 Jul 14:e76588. Online ahead of print. doi: 10.1002/advs.76588 (PMC13366363; doi:10.1002/advs.76588)
Supplement: Supplementary file 1 — Supporting File: advs76588‐sup‐0001‐SuppMat.pdf. [file ADVS-9999-e76588-s001.pdf]

Supplementary Information

Diastereomeric Monomers Enable Anion-Exchange Membranes with Controlled Local Polymer Backbone Flexibility and High Conductivity

Si Chen, Xuchen Lyu, Triet Nguyen Dai Luong, Patric Jannasch*

Department of Chemistry, Lund University, P.O. Box 124, SE-22100 Lund, Sweden

**Corresponding author:* patric.jannasch@chem.lu.se

Experimental descriptions and results

1. Materials and synthesis methods

1.1 Materials

Sodium borohydride (95%) was obtained from Riedel-de Haen. And TFSA (98%), 1-methyl-4- piperidone (98%), 4-acetylbiphenyl (98%) were purchased from TCI. Phosphorus tribromide (97%), TFA (ReagentPlus, 99%), potassium carbonate (ACS Reagent, 99%), dimethyl zinc solution (1.0 M in heptane), iodomethane (ReagentPlus, 99%) were obtained from Sigma-Aldrich, DMSO (99%), diethyl ether, isopropanol, dichloromethane (DCM), heptane, and HCl (37% in water) were obtained from Fisher. Potassium chromate solution (0.1 N) was obtained from Fluka Analytical. Dry dichloromethane and tetrahydrofuran were collected from an MBraun dry solvent dispenser system MB-SPS 800.

^{1}H and ^{13}C NMR spectra of monomers and precursors ($CDCl_3$, δ = 7.26 ppm, solution) and ^{1}H NMR spectra of polymers (DMSO-*d6*, δ = 2.50 ppm, solution) were recorded using a Bruker DRX 400 spectrometer. TFA was added to protonate any tertiary amines, enabling their detection and shifting the broad water signal to above 12 ppm to prevent signal overlap.

1.2 Synthesis of precursor 1

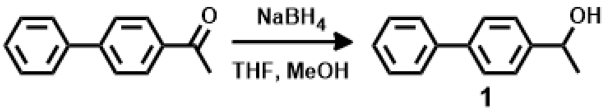

An amount of 10 g of 4-acetylbiphenyl was dissolved in a mixture of THF and methanol (200 mL, 1:4, v/v) in a 500 mL round-bottom flask equipped with an oval stir bar. The flask was cooled to 0 °C in an ice–water bath, and sodium borohydride (20 g, 10 equiv.) was added portion-wise over 1 h. The reaction mixture was allowed to warm to room temperature and stirred for an additional 2 h. The mixture was again cooled to 0 °C, and 2 M HCl (100 mL) was slowly added. The reaction was carried out in an open flask to avoid pressure buildup. The mixture was extracted with DCM (300 mL), and the organic layer was washed with deionized water (3 × 100 mL), dried over anhydrous Na_2SO_4, and concentrated under reduced pressure to afford a white solid (**1**) in quantitative yield. ^{1}H NMR (400 MHz, chloroform-*d*) δ 7.68 – 7.60 (m, 4H), 7.48 (td, *J* = 8.2, 1.9 Hz, 4H), 7.42 – 7.37 (m, 1H), 4.95 (q, *J* = 6.5 Hz, 1H), 2.43 (s, 1H), 1.57 (d, *J* = 6.5 Hz, 3H). ^{13}C NMR (101 MHz, chloroform-*d*) δ 144.94, 140.92, 140.41, 128.86, 127.34, 127.28, 127.15, 125.96, 70.14, 25.21.

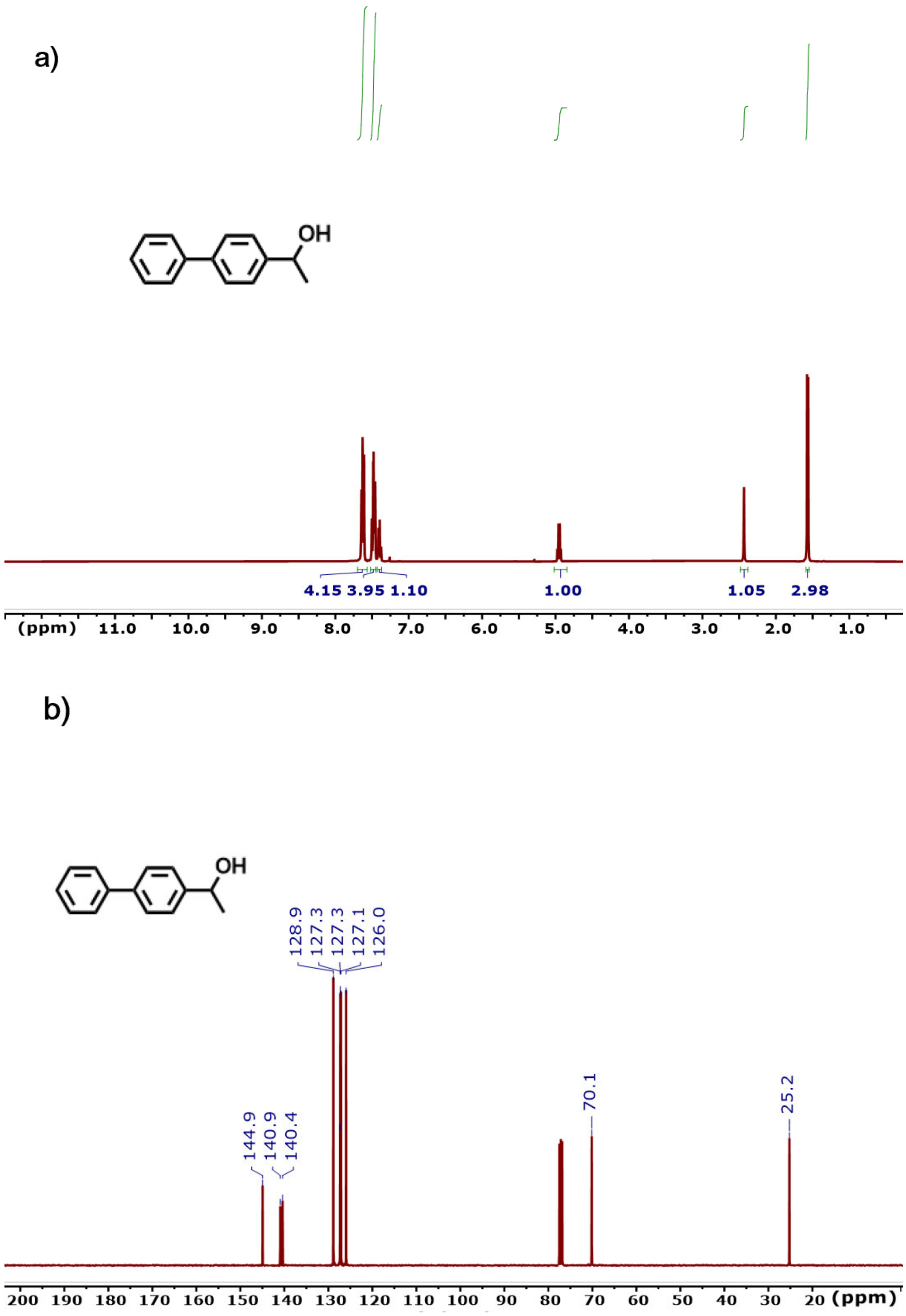

Figure S1. (a) ^{1}H NMR and (b) ^{13}C NMR spectra of precursor **1**.

1.3 Synthesis of precursor 2

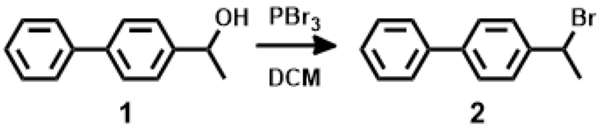

A 50 mL Schlenk tube equipped with an oval stir bar was charged with precursor **1** (10 g), sealed with a rubber septum, evacuated, and backfilled with nitrogen three times. Dry DCM (16 mL) was then added to dissolve precursor **1**, and the tube was cooled to 0 °C in an ice–water bath. Phosphorus tribromide (4.8 mL, 1 eq.) was added dropwise via a syringe. The reaction mixture was allowed to warm to room temperature and stirred overnight. The reaction mixture was then carefully poured into a cold saturated aqueous $NaHCO_3$ solution (200 mL). The resulting mixture was extracted with DCM (3 × 100 mL), and the combined organic layers were dried over anhydrous Na_2SO_4 and concentrated under reduced pressure to afford an off-white solid (**2**) in quantitative yield. ^{1}H NMR (400 MHz, chloroform-*d*) δ 7.63 – 7.57 (m, 4H), 7.56 – 7.51 (m, 2H), 7.49 – 7.43 (m, 2H), 7.41 – 7.35 (m, 1H), 5.30 (q, *J* = 6.9 Hz, 1H), 2.11 (d, *J* = 6.9 Hz, 3H). ^{13}C NMR (101 MHz, chloroform-*d*) δ 142.24, 141.34, 140.52, 128.86, 127.56, 127.45, 127.30, 127.14, 49.41, 26.78.

Note: Compound 2 rapidly decomposed on silica gel and could therefore not be purified by column chromatography.

a)

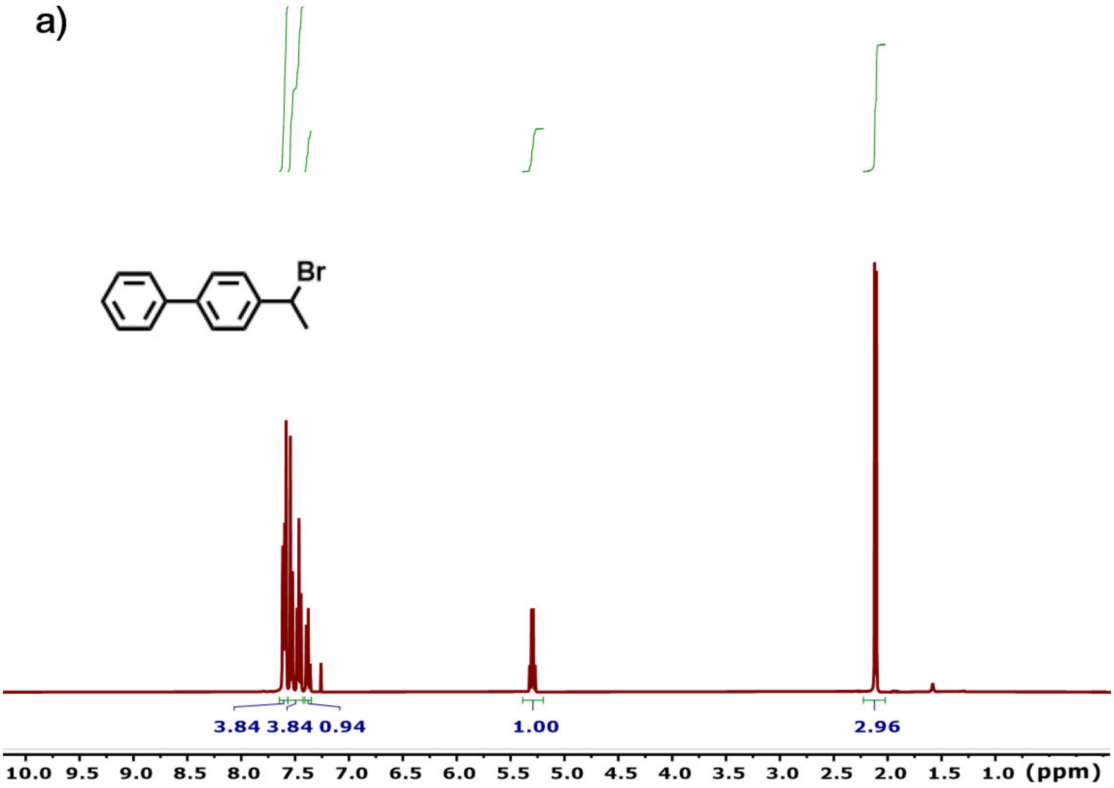

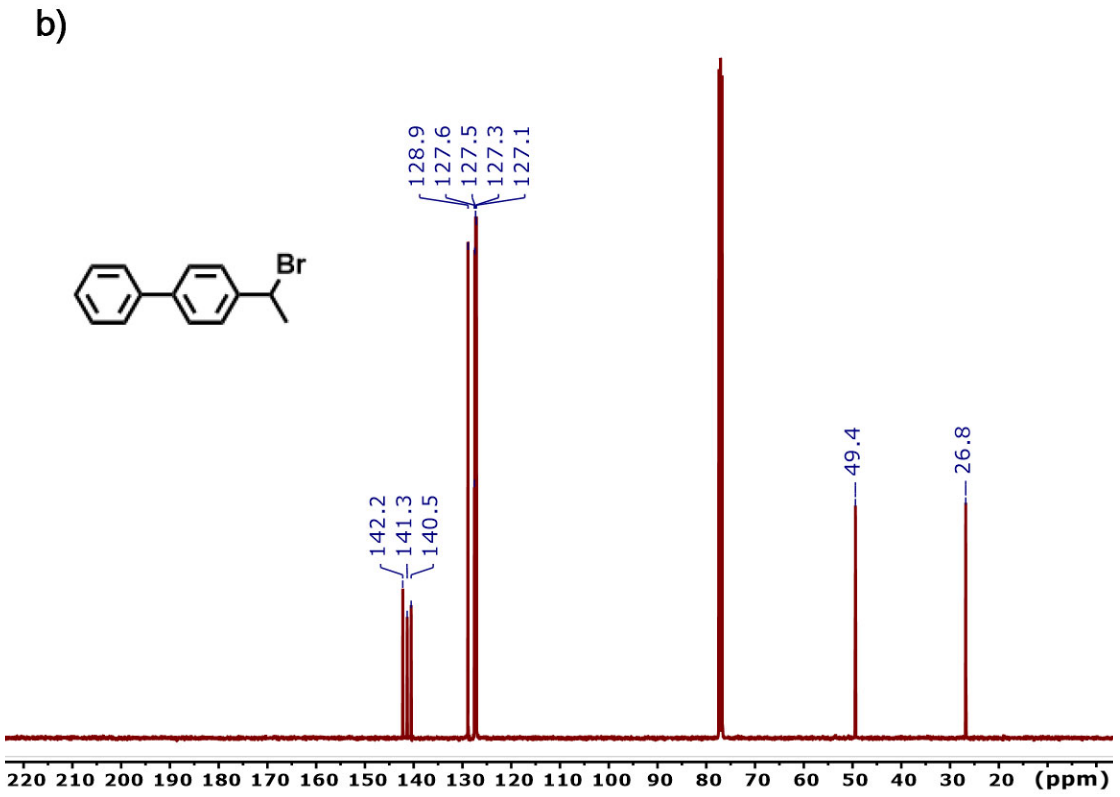

Figure S2. (a) ^{1}H NMR and (b) ^{13}C NMR spectra of precursor **2**.

1.4 Synthesis of the apD **and** *spD enriched monomers*

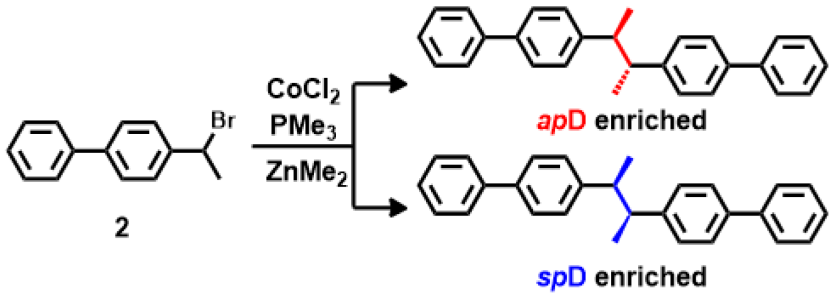

A 100 mL Schlenk flask equipped with an oval stir bar and sealed with a rubber septum was dried, evacuated, and backfilled with nitrogen three times. Under a nitrogen atmosphere, anhydrous $CoCl_2$ (52 mg, 2 mol%) was added, followed by dry THF (16 mL). After adding the trimethylphosphine (1.0 M in THF, 1.6 mL, 4 equiv relative to $CoCl_2$), the solution turned black. Dimethylzinc (1.0 M in heptane, 12 mL, 0.6 equiv relative to compound 2) was subsequently added, giving a dark red solution. Compound 2 (5.2 g, 20 mmol) was dissolved in dry THF (10 mL) and rapidly added to the reaction mixture. The flask was immediately placed in a preheated

sand bath at 66 °C. After 20 min, the reaction was quenched by cooling to 0 °C, followed by the addition of 2 M aqueous HCl (6 mL). The resulting white precipitate was collected by filtration and washed with cold heptane to afford the *ap*D-enriched monomer as a white solid. The filtrate was diluted with DCM (200 mL) and washed with deionized water (3 × 100 mL). Next, the organic layer was dried over anhydrous Na_2SO_4 and concentrated under reduced pressure. The residue was purified by column chromatography (DCM/heptane = 1:8, v/v) to afford the *sp*D-enriched monomer as a white solid. The overall isolated yield was approximately 76%.

*ap*D-enriched monomer:

^{1}H NMR (400 MHz, chloroform-*d*) δ 7.60 (dd, *J* = 22.4, 7.7 Hz, 8H), 7.45 (t, *J* = 7.5 Hz, 4H), 7.33 (dd, *J* = 13.4, 7.5 Hz, 6H), 2.89 (d, *J* = 7.5 Hz, 2H), 1.12 (d, *J* = 5.8 Hz, 6H).

^{13}C NMR (101 MHz, chloroform-*d*) δ 145.57, 141.09, 138.98, 128.73, 128.07, 127.02, 127.00, 46.93, 21.08.

*sp*D-enriched monomer:

^{1}H NMR (400 MHz, chloroform-*d*) δ 7.62 – 7.57 (m,4H), 7.49 – 7.40 (m, 8H), 7.36 – 7.30 (m, 2H), 7.19 – 7.13 (m, 4H), 3.13 – 3.02 (m, 2H), 1.39 – 1.34 (m, 6H).

^{13}C NMR (101 MHz, chloroform-*d*) δ 144.87, 141.04, 138.56, 128.67, 128.33, 126.93, 126.51, 46.03, 17.88.

1.5 Polymerization

The polymerization affording sample P*ap*DPip-25 is given as a representative example. To a 10 mL round-bottom flask equipped with an oval magnetic stir bar were added the *ap*D-enriched monomer (91 mg, 0.25 mmol), *p*-terphenyl (173 mg, 0.75 mmol), and *N*-methyl-4-piperidone (130 mg, 1.15 mmol), followed by DCM (0.7 mL). The mixture was cooled to 0 °C in an ice bath, before adding trifluoroacetic acid (TFA, 0.15 mL). Trifluoromethanesulfonic acid (TFSA or TfOH, 1.0 mL, 11.3 mmol) was then added dropwise. The polymerization was allowed to proceed at room temperature for 11 h (the reaction time was 27 h for P*ap*DPip-70 and 17 h for P*sp*DPip-40). Subsequently, dimethyl sulfoxide (DMSO, 6 mL) was added to dilute the viscous reaction mixture. The resulting solution was poured into a 1:1 (v/v) mixture of diethyl ether and isopropanol (100 mL) to precipitate the polymer as a white powder. The polymer was collected by filtration, washed with water, and dried in a vacuum oven at 50 °C to afford P*ap*DPip-25 in 87% yield.

The amounts of *N*-methyl-4-piperidone, DCM, TFA, and TFSA were kept the same for all polymerizations.

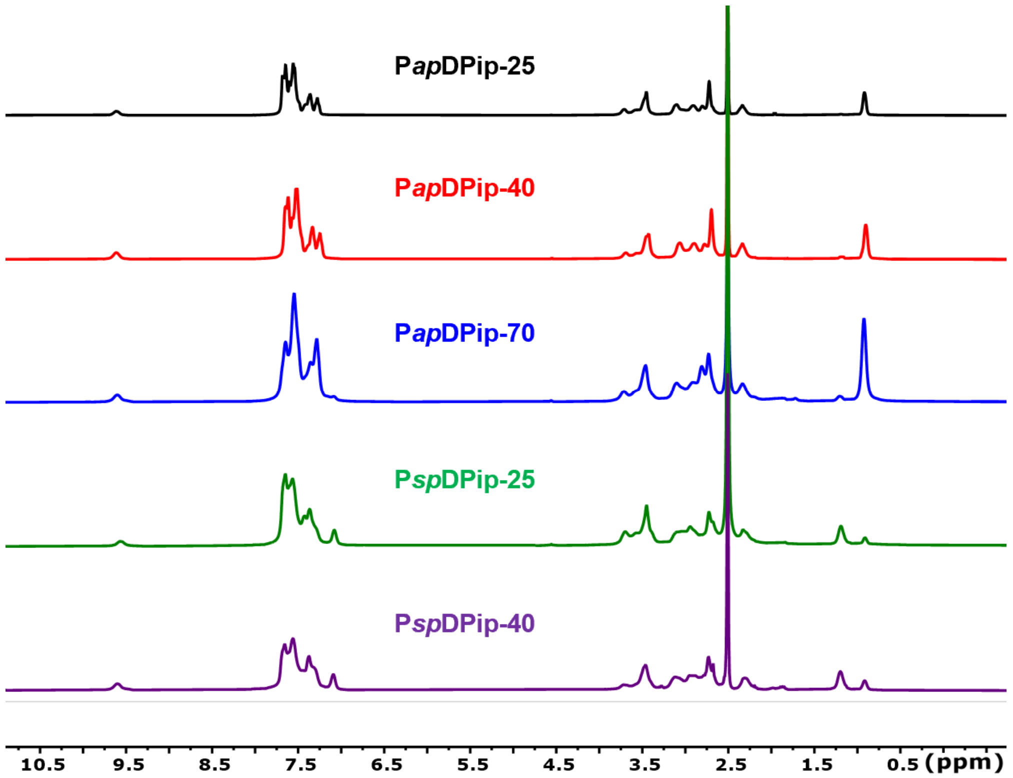

Figure S3. ^{1}H NMR spectra of the P*ap*DPip-*x* and P*sp*DPip-*x* precursor polymers recorded in DMSO-d_6 solutions (TFA was added to shift the water signal).

1.6 Quaternization

The synthesis of P*ap*DPipQ-25 is provided as a representative example of the quaternization reaction. To a 25 mL round-bottom flask equipped with a magnetic stir bar were added P*ap*DPip-25 (300 mg, corresponding to 0.82 mmol of *N*-methylpiperidine units), K_2CO_3 (222 mg, 1.6 mmol), and methyl iodide (0.15 mL, 2.4 mmol, 2.93 equiv), followed by dimethyl sulfoxide (DMSO, 8 mL). The flask was sealed and protected from light by aluminum foil. After stirring for 48 h, the reaction mixture was poured into a 1:1 (v/v) mixture of diethyl ether and isopropanol (100 mL) to precipitate the product. The polymer was collected by filtration, washed with a mixture of isopropanol and water, and dried under vacuum to afford P*ap*DPipQ-25 as an off-white solid in quantitative yield.

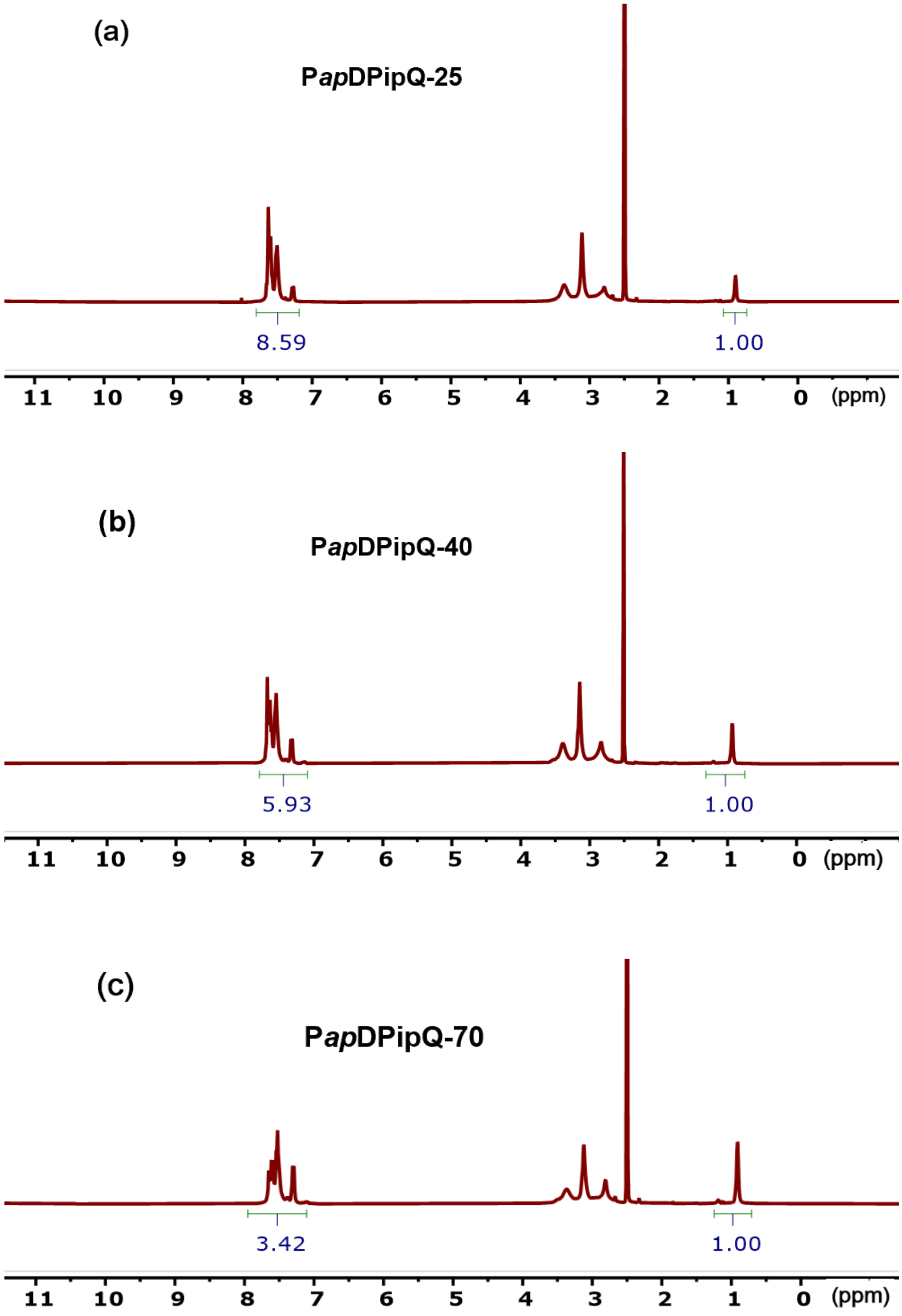

(a)
PapDPipQ-25
8.59
1.00
11 10 9 8 7 6 5 4 3 2 1 0 (ppm)
(b)
PapDPipQ-40
5.93
1.00
11 10 9 8 7 6 5 4 3 2 1 0 (ppm)
(c)
PapDPipQ-70
3.42
1.00
11 10 9 8 7 6 5 4 3 2 1 0 (ppm)

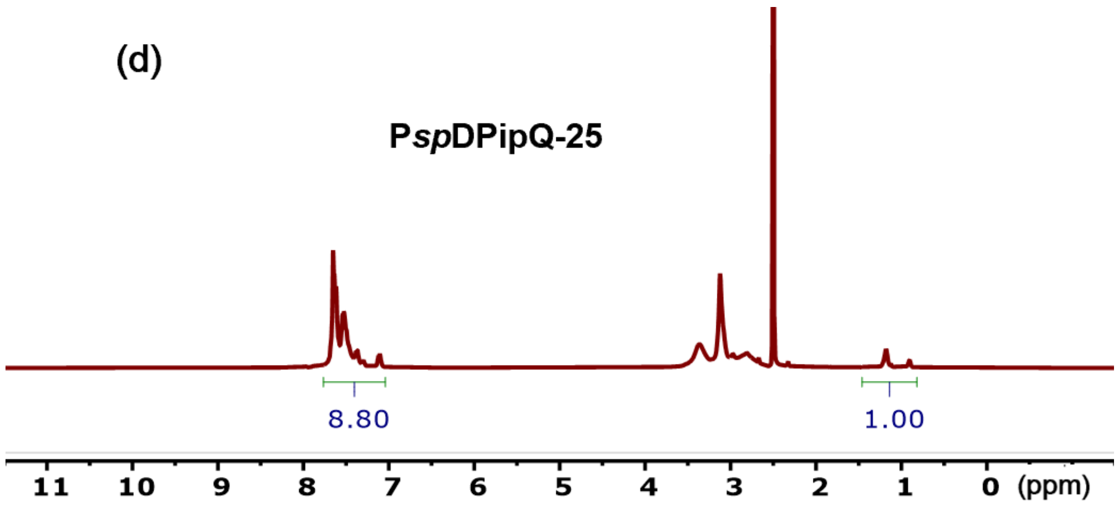

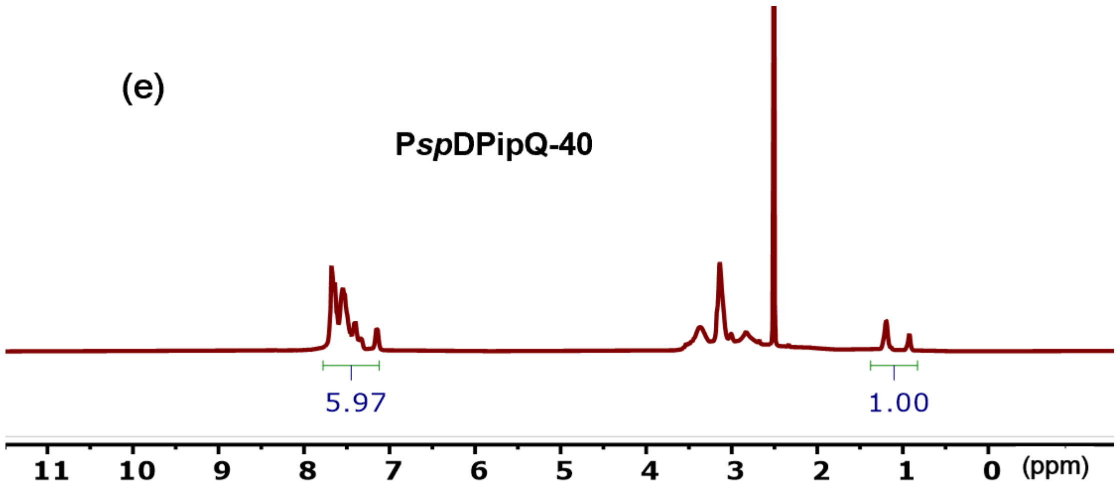

Figure S4. ^{1}H NMR spectra of a) P*ap*DPip-25, b) P*ap*DPip-40, c) P*ap*DPip-70, d) P*sp*DPip-25 and e) P*sp*DPip-40 recorded using DMSO-d_6 solutions (TFA was added to shift the water signal).

Table S1. Molar monomer feed ratios and copolymer contents of *ap*D or *sp*D relative to *p*-terphenyl in P*ap*DPipQ-*x* and P*sp*DPipQ-*x*, expressed as *ap*D : *p*-terphenyl or *sp*D : *p*-terphenyl ratios, respectively, determined by ^{1}H NMR and titration data.

AEM	Feed ratio (to *p*-terphenyl) (%)	Calculated from NMR results (%)	Calculated from titration results (%)
P*ap*DPipQ-25	25 : 75	25 : 75	24 : 76
P*ap*DPipQ-40	50 : 50	38: 62	37 : 63
P*ap*DPipQ-70	75 : 25	73 : 27	69 : 31
P*sp*DPipQ-25	25 : 75	25 : 75	26 : 74
P*sp*DPipQ-40	40 : 60	38 : 62	41 : 59

2. DFT calculations of structures and bond rotations for the *ap*D and *sp*D units

All calculations were performed using the ORCA 5.0 software. A global conformational search was performed to identify the optimized geometry for *ap*D and *sp*D, respectively. The restrictions on the dihedral angle [C(CH3)-C-C-C(CH3)] were imposed to conduct the structural optimization of 72 conformers (360° /5°, 72 conformers). These conformers were optimized using the wB97X-D function with the DEF2TZVP basis set, and the energy was calculated using the wB97X-D function with the DEF2TZVP basis set.

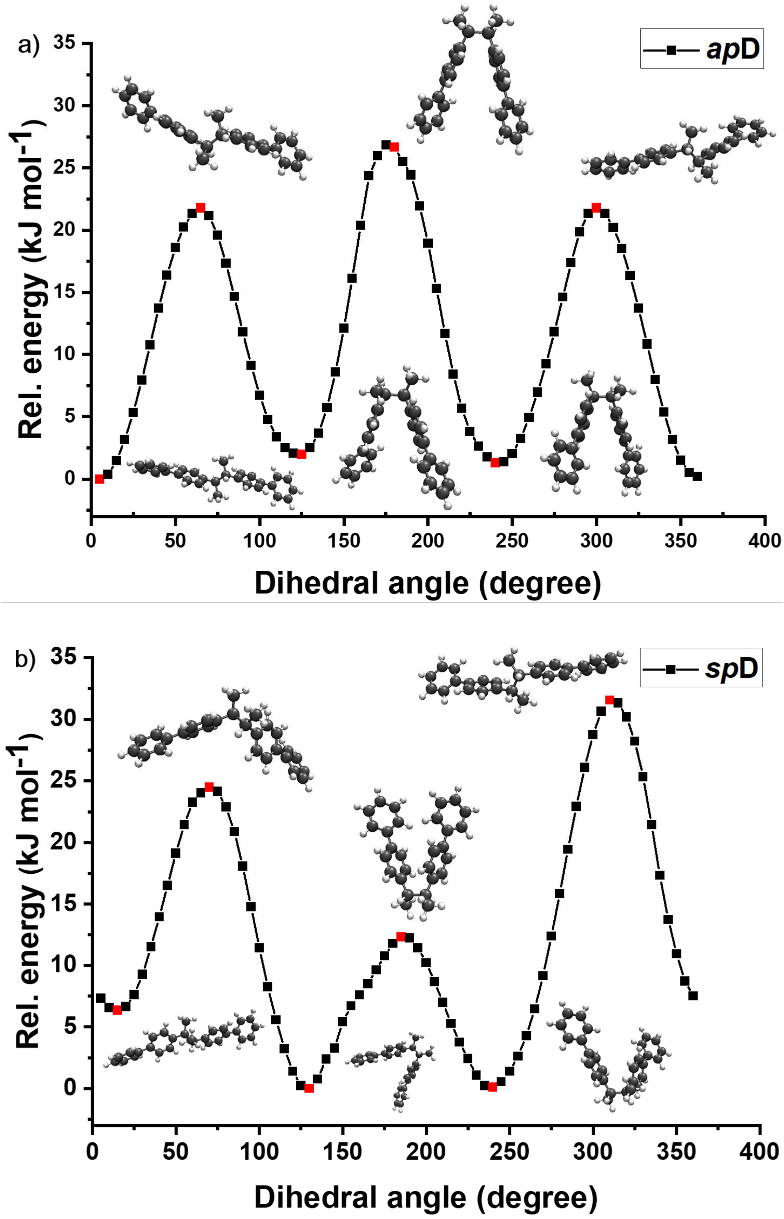

Figure S5. Potential energy profiles for the flexible scan of the [C(CH3)-C-C-C(CH3)] torsion angle (rotation of the C-C bond) in the a) *ap*D, and b) *sp*D structures.

3. Monomer crystallization

DSC analysis was carried out with a TA Instruments DSC Q2000 differential scanning calorimeter. An amount of 6 mg of the dried *ap*D- and *sp*D-enriched monomers was first heated to 260 °C or 170 °C respectively at 10 °C min^{-1}, The samples were then maintained at this temperature for 5 min, before cooling to –50 °C at a rate of 10 °C min^{-1}, followed by a 5 min isothermal period. Finally, the samples were heated to the starting temperature at the same rate.

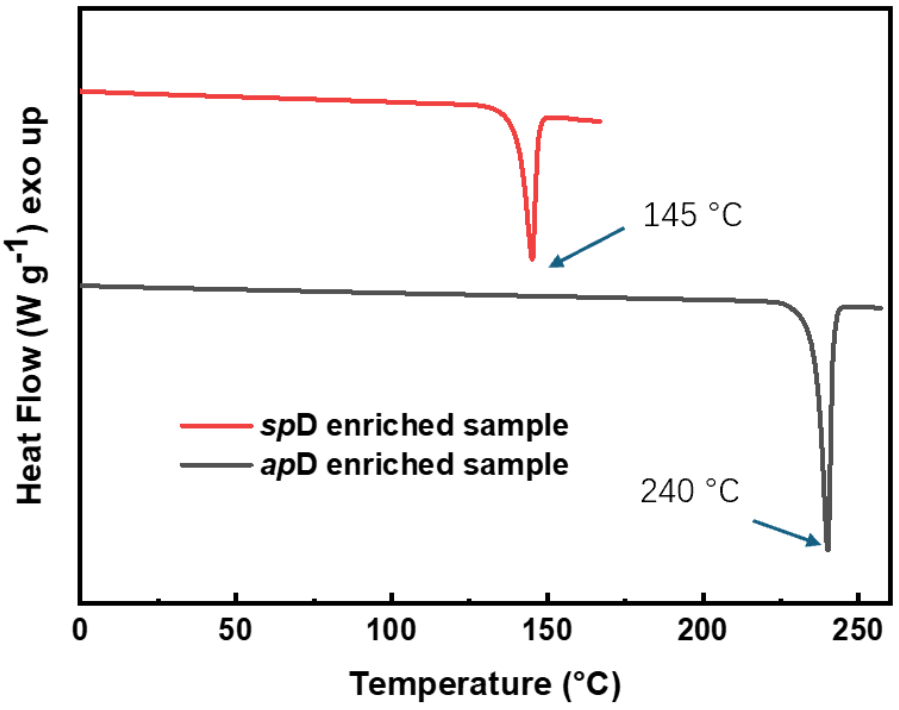

Figure S6. DSC traces recorded during the second heating scan of the *ap*D- and *sp*D-enriched monomer samples.

4. Intrinsic viscosity

The intrinsic viscosity of all the P*ap*DPipQ-*x* and P*sp*DPipQ-*x* samples was measured at 25 °C using an Ubbelohde viscosimeter. The AEMs were weighed and dissolved in 0.1 M LiBr in DMSO (to prevent the polyelectrolyte effect) to obtain stock solutions with a concentration of 1 g dL^{-1}. A solution of 0.1 M LiBr in DMSO was prepared as the blank. The stock solutions were subsequently diluted by adding blank solution to obtain four different polymer concentrations (C). These solutions were kept at 25 °C in a water bath for 20 minutes equilibration before loaded into the Ubbelohde viscosimeter. The flow times of the blank solution (t_{blank}) and the different polymer solutions (t_{sample}) through the capillary were taken as the average of three measurements. The reduced (η_{red}) and inherent (η_{inh}) viscosities at the four different concentrations were calculated as shown in Equation S1 and S2, respectively. The

intrinsic viscosity was obtained by plotting η_{red} as function of polymer concentration, followed by linear regression and extrapolation to $C = 0$ g dL^{-1}.

$$\eta_{\mathrm{red}} = \frac{\frac{t_{\mathrm{sample}}}{t_{\mathrm{blank}}} - 1}{C} \quad \text{(Eq. S1)}$$

$$\eta_{\mathrm{inh}} = \frac{\ln\left(\frac{t_{\mathrm{sample}}}{t_{\mathrm{blank}}}\right)}{C} \quad \text{(Eq. S2)}$$

5. Mechanical properties

Mechanical properties of the AEMs were evaluated by measuring the stress-strain data of dry AEM samples in the bromide form using a TA Instruments Q800 dynamic mechanical analysis (DMA) instrument. Rectangular samples (approx. 0.6 cm × 2.8 cm, 60 μm in thickness) were prepared and mounted between the tension clamps before applying a 0.01 N preload force. The measurements were then conducted with a ramping force of 0.5 N min^{-1} at 27 °C in a controlled force mode.

6. Morphology

6.1 Small angle X-ray scattering (SAXS)

SAXS measurements were carried out on a SAXLAB instrument (JJ X-ray Systems ApS, Denmark) equipped with a Pilatus detector. Before the measurement, AEM samples were ion-exchange to Br^{-} form and were dried for 48 h. The thickness of membranes was around 60 μm.

6.2 Atomic Force Microscopy (AFM)

A Bruker Icon Atomic Force Microscope instrument with TESPAV2 tips was used to collect images. Tapping mode was used to record the phase image of the AEMs (with Br^{-} as counter ion) in ambient atmosphere.

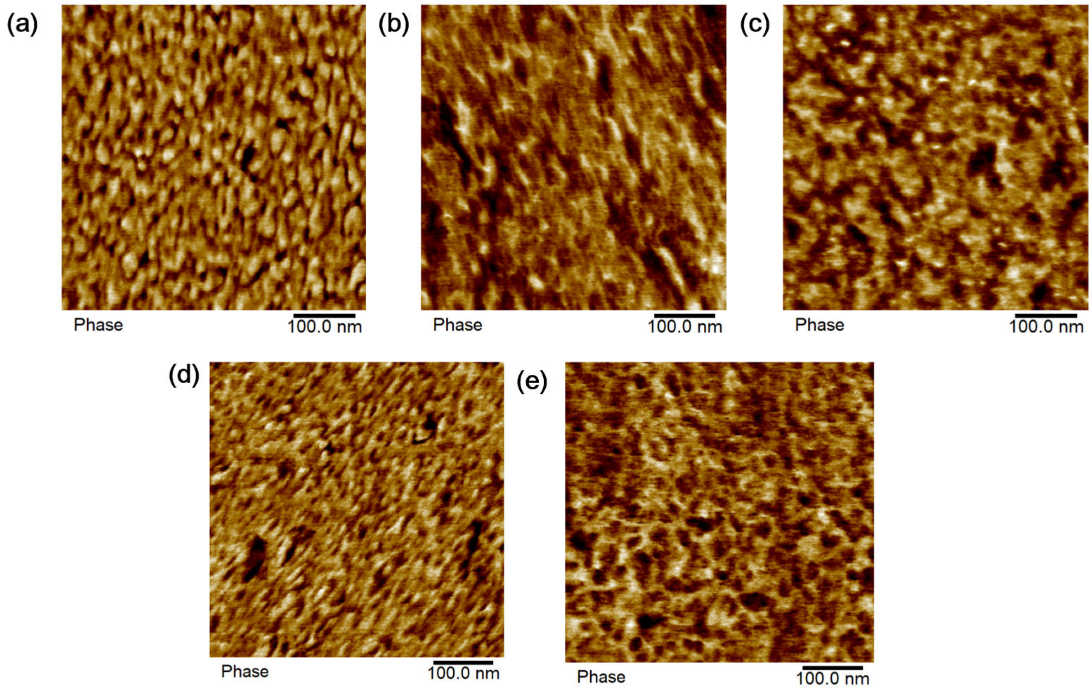

Figure S7. AFM phase images of a) P*ap*DPipQ-25, b) P*ap*DPipQ-40, c) P*ap*DPipQ-70, d) P*sp*DPipQ-25 and e) P*sp*DPipQ-40.

7. Ion exchange capacity, water and electrolyte uptake, and swelling ratio

The ion exchange capacity (IEC) of the AEMs was determined by Mohr titrations. Around 30 mg of each sample in the Br^- form was dried under vacuum at 50 °C for at least 48 h. These samples were precisely weighed before immersion into 25 mL 0.2 M aq. $NaNO_3$ at 40 °C for 48 h. After the complete ion exchange, the resulting solution was titrated with 0.01 M aq. $AgNO_3$ using K_2CrO_4 as the color indicator. Four titrations were carried out on 5 mL portions for each sample. The average result of the four measurements was used to calculate IEC in the OH^- form using the following equation:

$$IEC_{\mathrm{OH}} = \frac{IEC_{\mathrm{Br}}}{1 - 0.0629 \times IEC_{\mathrm{Br}}} \qquad \text{(Eq. S3)}$$

The uptake of pure water and 2 M aq. KOH solution of the AEMs in the OH^- form was measured gravimetrically. Dry samples in the Br^- form were weighed ($W_{\mathrm{dry,\,Br}}$) and then ion-exchanged to the OH^- form in 1 M aq. KOH for 48 h. The samples were then washed and stored in fresh degassed DI water or 2 M aq. KOH at 20, 40, 60 and 80 °C, respectively. After 24 h equilibration at 20 °C, and 8 h at the higher temperatures, the sample was taken out and quickly wiped dry with tissue paper and weighed ($W_{\mathrm{wet,\,OH}}$). Finally, the uptake was calculated with $W_{\mathrm{dry,\,OH}}$ obtained using the titrated IEC_{Br}:

$$W_{\text{dry,OH}} = W_{\text{dry,Br}} \times (1 - 0.0629 \times IEC_{\text{Br}}) \qquad \text{(Eq. S4)}$$

$$WU = \frac{W_{\text{wet,OH}} - W_{\text{dry,OH}}}{W_{\text{dry,OH}}} \times 100\% \qquad \text{(Eq. S5)}$$

Along with the uptake measurements, the dimensional changes in length (l) and thickness (t) of the AEMs were recorded to evaluate the in- (SW_i) and through-plane (SW_{th}) swelling at different temperatures. A micrometer screw gauge and a Vernier scale were used for the measurements. The swelling ratios were then calculated as:

$$SW_{\text{i}} = \frac{l_{\text{wet}} - l_{\text{dry}}}{l_{\text{dry}}} \times 100\% \qquad \text{(Eq. S6)}$$

$$SW_{\text{th}} = \frac{t_{\text{wet}} - t_{\text{dry}}}{t_{\text{dry}}} \times 100\% \qquad \text{(Eq. S7)}$$

8. Conductivity measurements

The in-plane OH^- conductivity of fully hydrated AEMs immersed in degassed DI water was measured by electrochemical impedance spectroscopy (EIS) between 20 and 80 °C. Square-shaped AEM samples (1.4 × 1.4 cm^2) in the Br^- form were prepared and immersed in a 1 M aqueous KOH at room temperature for 48 h. The alkaline solution was replaced 3 times with freshly prepared KOH solution during the storage to ensure complete ion exchange. Subsequently, the ion-exchanged samples were thoroughly washed with degassed DI water and stored in a N_2-ventilated desiccator to prevent CO_2 contamination. The thicknesses of the membranes were measured after equilibration in water at room temperature, before the assembly of the test cell. The measurements were conducted using a 2-probe cell mounted in a Novocontrol high-resolution dielectric analyzer V 1.01S, operating at an amplitude of 10 mV between 10^7–10^0 Hz.

9. Stability

9.1 The thermal stability

The thermal stability of the AEMs in the Br^- form and the non-quaternized precursors were evaluated by thermogravimetric analysis (TGA) using a TA Instruments TGA Q500 under N_2 atmosphere. For each sample, a preheating process at 150 °C for 20 min were performed to remove any liquid residues before heating from 50 to 600 °C at a rate of 10 °C min^{-1}. The temperature at 5% weight loss was reported as the decomposition temperature ($T_{d,95}$).

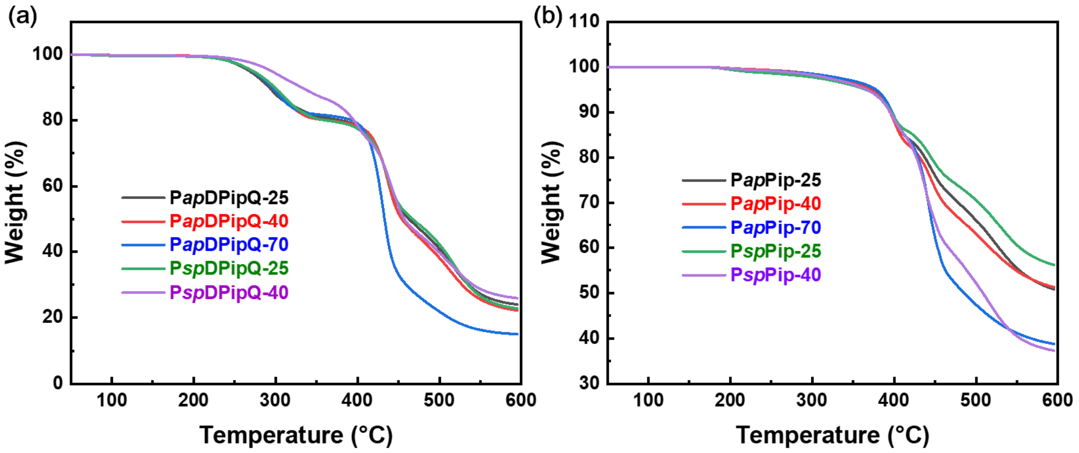

Figure S8. TGA traces of the a) P*ap*DPipQ-*x* and P*sp*DPipQ-*x*, b) P*ap*DPip-*x* and P*sp*DPip-*x* series of AEMs.

9.2 Alkaline stability

AEM samples were placed in sealed pressure-resistant Teflon-coated tubes containing 2 M aq. KOH, which were then stored at 90 °C for 720 h. After predetermined storage times, samples were taken out and ion-exchanged to the Br− form for 48 h. Then, the samples were washed with DI water and dried. ^{1}H NMR spectroscopy was then performed on these samples using DMSO-*d6* as solvent with 5-10 vol% TFA added.

10. AEMWE performance and durability test

The AEMWE set-up was assembled according to a reported method.[1] A 2.5 × 2.5 cm active area test cell was used to evaluate the AEMWE performance. The nickel foam electrode material was purchased from Redox Flow, Denmark. The 56-63 μm-thick AEMs P*ap*DPipQ-40, P*ap*DPipQ-70, P*sp*DPipQ-40, and a 60 μm-thick commercial benchmark AEM (PiperION® 60) were evaluated in the cell. A 2 M aq. KOH electrolyte solution was circulated and fed into both the anode and the cathode with a flow rate of 2 mL min^{-1}. The cell was assembled at a torque of 6 Nm. Polarization curves were obtained by conducting Staircase Galvanostatic Electrochemical Impedance Spectroscopy (SGEIS) measurements using 100 mA stepwise increases in the current. EIS measurements were conducted in galvanocontrol (GEIS) modes at frequencies of 0.1 Hz to 100 kHz at a current density of 32 mA/cm^2. The long-term stability test was conducted at a constant current density of 300 mA cm^{-2} with electrolyte changes every day. All electrochemical measurements were conducted using a VSP potentiostat (Bio-Logic, France).

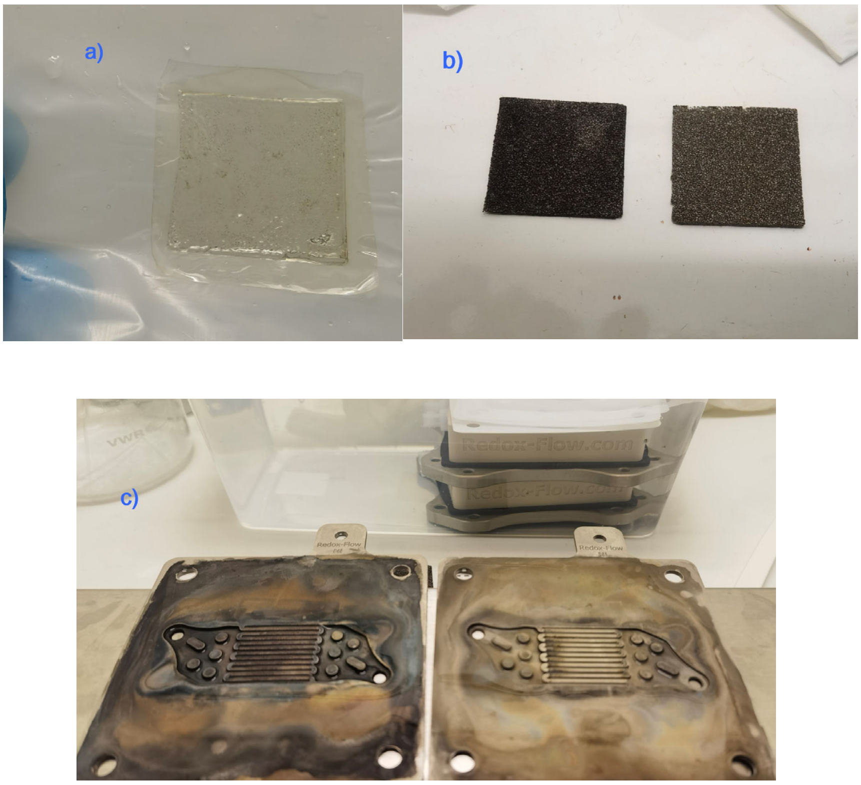

Figure S9. Photographic images of a) P*ap*DPipQ-40, b) the nickel foam electrodes, and c) the current collectors after 200 h AEMWE operation in aq. 2 M KOH at 80 °C.

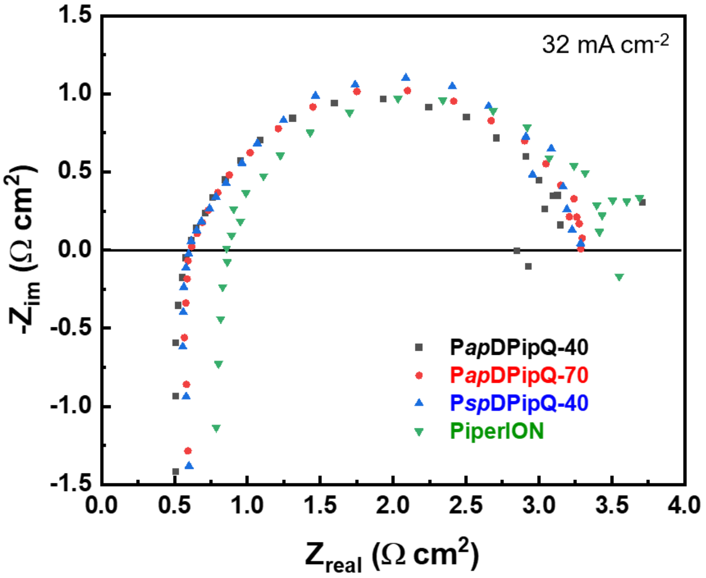

Figure S10. Galvanostatic electrochemical impedance spectra (GEIS) of P*ap*DPipQ-40, P*ap*DPipQ-70, P*sp*DPipQ-40, and PiperION ® 60, recorded at 80 °C, with 2 M aq. KOH solution using nickel foam electrodes. The ohmic resistance was estimated from the high-frequency intercept with the Zreal axis, and the charge-transfer resistance was estimated from the diameter of the semicircle.

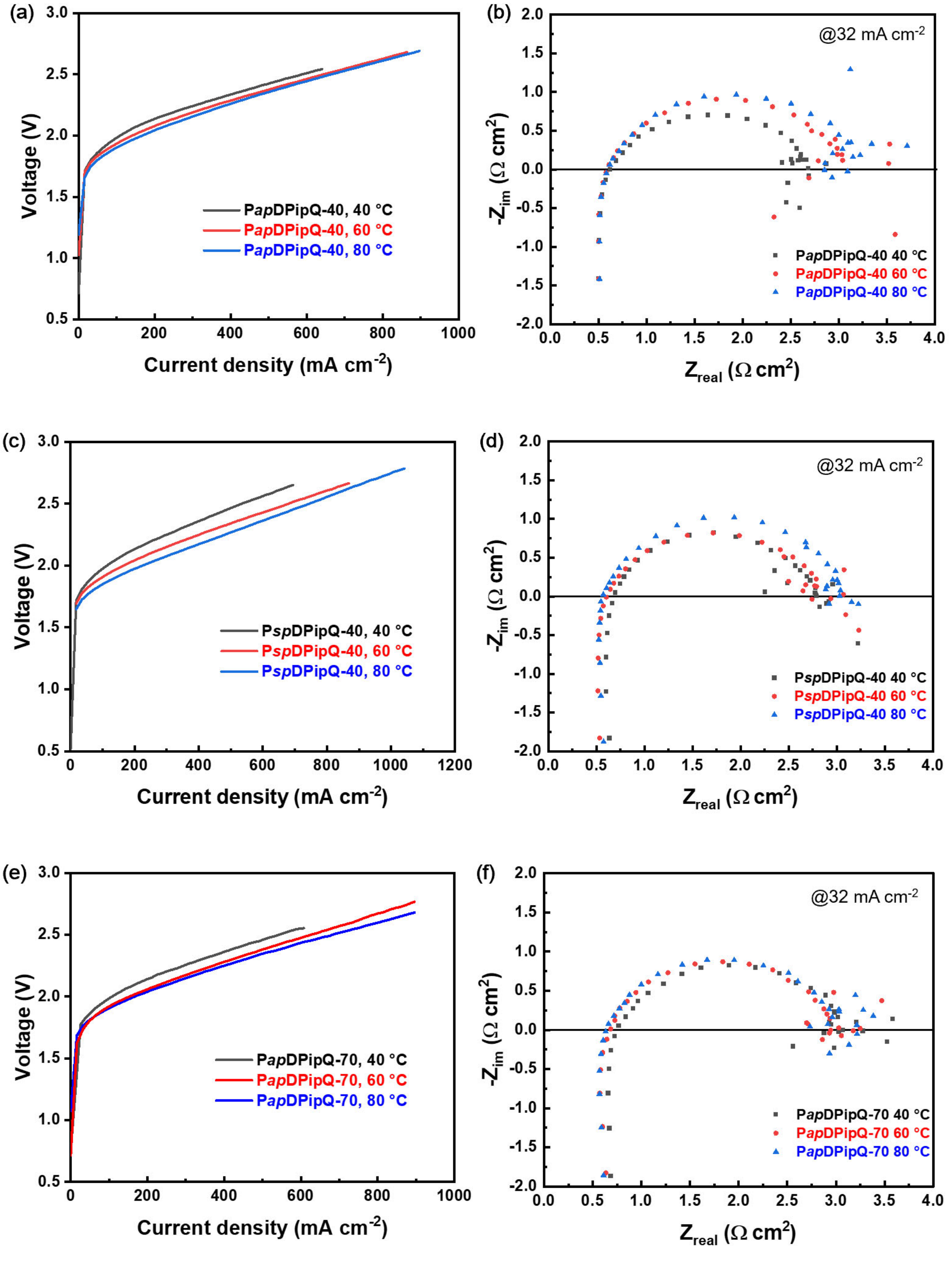

Figure S11. Polarization curves of (a) P*ap*DPipQ-40, (c) P*sp*DPipQ-40, and (e) P*ap*DPipQ-70, measured with 2 M aq. KOH at 40, 60, and 80 °C, respectively, and corresponding GEIS of (b) P*ap*DPipQ-40, (d) P*sp*DPipQ-40, and (f) P*ap*DPipQ-70 in 2 M KOH at 40, 60 and 80 °C (measured at 32 mA cm^{-2}).

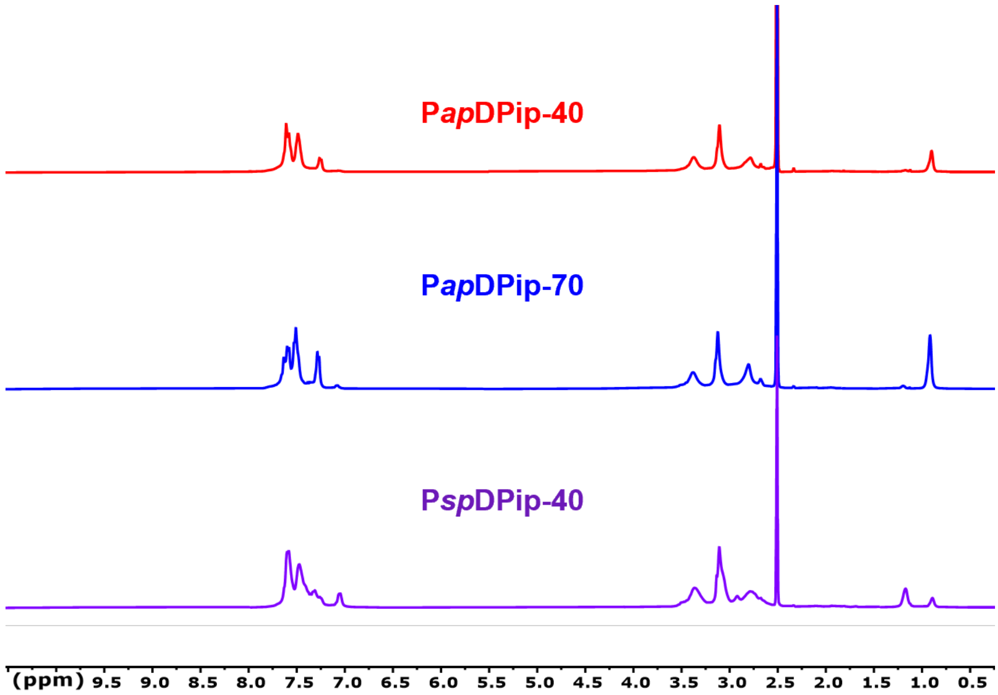

Figure S12. ^{1}H NMR spectra of P*ap*DPipQ-40, P*ap*DPipQ-70, and P*sp*DPipQ-40 after 200 h operation of AEMWE at 300 mA and 80 °C with 2 M aq. KOH solution.

11. Statistical analysis

No statistical hypothesis testing was performed in the present study, and no probability values or significance symbols were used in the work. Unless otherwise stated, the data are presented as experimentally measured values. No data points were excluded from the analysis. Data processing, including spectral integration, baseline correction, normalization, and fitting, where applicable, was carried out as described in the corresponding experimental sections. Figures were prepared using OriginPro® and Microsoft Excel®.

Reference

[1] T. N. D. Luong, P. Jannasch, Incorporating chrysene units to improve the performance of poly(arylene piperidinium) and poly(arylene quinuclidinium) anion exchange membranes for water electrolysis. *Journal of Materials Chemistry A*, **2026**, *14*, 16796-16806. DOI: 10.1039/D5TA10393B
